# Identification of novel growth factor-responsive genes in neuroendocrine gastrointestinal tumour cells

**DOI:** 10.1038/sj.bjc.6602535

**Published:** 2005-04-20

**Authors:** E Hofsli, L Thommesen, F Yadetie, M Langaas, W Kusnierczyk, U Falkmer, A K Sandvik, A Laegreid

**Affiliations:** 1 Department of Cancer Research and Molecular Medicine, Faculty of Medicine, Norwegian University of Science and Technology, Medisinsk Teknisk Forskningssenter, Trondheim N-7489, Norway; 2 Oncology Unit, St Olavs Hospital HF, Trondheim, Norway; 3 Department of Mathematical Sciences, Norwegian University of Science and Technology, Trondheim, Norway; 4 Department of Computer and Information Science, Norwegian University of Science and Technology, Trondheim, Norway

**Keywords:** neuroendocrine tumours, growth factors, proliferation, gene expression profiling, gastrin, hepatocyte growth factor

## Abstract

Targeting growth-regulatory pathways is a promising approach in cancer treatment. A prerequisite to the development of such therapies is characterisation of tumour growth regulation in the particular tumour cell type of interest. In order to gain insight into molecular mechanisms underlying proliferative responses in neuroendocrine (NE) gastrointestinal (GI) tumours, we investigated gene expression in human carcinoid BON cells after exposure to gastrin, hepatocyte growth factor (HGF), pituitary adenylate cyclase-activating polypeptide or epidermal growth factor. We particularly focused on gastrin- and HGF-induced gene expression, and identified 95 gastrin- and 101 HGF-responsive genes. The majority of these genes are known mediators of processes central in tumour biology, and a number of them have been associated with poor prognosis and metastasis in cancer patients. Furthermore, we identified 12 genes that were regulated by all four factors, indicating that they may be universally regulated during NE GI tumour cell proliferation. Our findings provide useful hypotheses for further studies aimed to search for new therapeutic targets as well as tumour markers in NE GI tumours.

Carcinoids are the most common type of neuroendocrine (NE) tumours found in the gastrointestinal (GI) system (Öberg, 2000; Modlin *et al*, 2003). Although rare, with an incidence of approximately 1–2/100 000 per year (Öberg, 2000; Modlin *et al*, 2003), the prevalence is high because of their usually well-differentiated nature and relatively slow growth rate. However, some patients have highly aggressive tumours leading to early death, while many of those living for several years have debilitating symptoms due to hypersecretion of biogenic amines and hormones. Surgery is the only potentially curative treatment, but since distant metastasis or regional disease are often present at the time of diagnosis, cure is most often not possible (Modlin *et al*, 2003). Thus, new therapeutic options are needed. Targeting key growth-regulatory pathways is a promising approach in cancer treatment (Behe and Behr, 2002; Yee, 2002; Sridhar *et al*, 2003; Ranson, 2004; Jiang *et al*, 2005), and requires a thorough knowledge of molecular mechanisms involved in growth regulation of various tumour cell types.

The amidated form of the peptide hormone gastrin is a well-known regulator of gastric acid secretion and GI mucosal growth and organisation (Waldum *et al*, 1995; Dockray *et al*, 2001, 2005). In addition, there is abundant evidence to suggest that gastrin may play an important role in tumour biology, as it is shown to regulate tumour cell growth (Dockray *et al*, 2001, 2005), and stimulate tumour cell invasion (Kucharczak *et al*, 2001) as well. Furthermore, hypergastrinaemia is associated with the occurrence of gastric enterochromaffin-like (ECL) cell carcinoid tumours (Waldum *et al*, 1995; Dockray *et al*, 2001, 2005; Richards *et al*, 2004) and with an increased risk of gastric and colorectal carcinoma (Dockray *et al*, 2001), indicating a role also in carcinogenesis. The biological actions of amidated gastrin are exerted through high-affinity binding to the gastrin/cholecystokinin B (CCK2) receptor (Denyer *et al*, 1994). This receptor has been shown to be coexpressed with gastrin in several GI tumour cell lines (Watson *et al*, 1998) and in human gastric carcinoids (Smith *et al*, 1998), indicating the existence of an autocrine gastrin growth-stimulatory loop. Interestingly, progress is being made in the development of CCK2 receptor targeting peptides for staging and therapy of human tumours (over)expressing this receptor (Behe and Behr, 2002).

Hepatocyte growth factor (HGF)/scatter factor was originally described as a factor promoting growth and scattering of multiple myeloma cells (Børset *et al*, 1996; Maulik *et al*, 2002). It is now clear that HGF exerts pleiotropic effects in a wide variety of tumour cells, where binding of HGF to its tyrosine kinase receptor c-Met promotes scattering, proliferation, angiogenesis, enhanced cell motility, invasion and metastasis (Maulik *et al*, 2002). However, its role in NE GI tumour cell biology is not yet defined. The same is true concerning the pleiotropic hormone pituitary adenylate cyclase-activating polypeptide (PACAP), which is known to regulate proliferation, differentiation and apoptosis in a number of malignant cells (Sherwood *et al*, 2000). However, PACAP and its various receptor forms have been found expressed in human NE tumours such as pheochromocytomas and neuroblastomas (Reubi *et al*, 2000; Sherwood *et al*, 2000), and PACAP has been found to modulate rat gastric ECL cell proliferation (Läuffer *et al*, 1999), indicating a possible role of PACAP in NE GI tumour cell biology as well.

Targeting the epidermal growth factor (EGF) receptor has emerged as a promising approach in the treatment of various types of cancers (Sridhar *et al*, 2003; Ranson, 2004). The EGF receptor is found (over)expressed in various human NE tumours (Nilsson *et al*, 1995; Wulbrand *et al*, 1998; Peghini *et al*, 2002), including GI carcinoids. More recently, the EGFR tyrosine kinase inhibitor gefitinib has been shown to inhibit growth of various NE GI tumour cells, including human carcinoid BON cells (Höpfner *et al*, 2003). The establishment of the BON cell line has been a major contribution to the study of growth regulation of NE GI tumours (Evers *et al*, 1991). Both serotonin (Ishizuka *et al*, 1992) and insulin-like growth factor (IGF)-1 (von Wichert *et al*, 2000) have been shown to act as autocrine regulators of growth of BON cells, while nerve growth factor (Bold *et al*, 1995) and basic fibroblast growth factor (FGF) stimulate growth in a nonautocrine manner (Townsend *et al*, 1993).

Various growth factor receptors are attractive targets for novel treatment approaches in NE GI tumour disease. In the present study, we employ transcript profiling to examine molecular mechanisms underlying proliferative responses in BON carcinoid tumour cells after exposure to gastrin, HGF, EGF and PACAP. By this approach, we identify a number of genes not previously known to be regulated by these growth factors and discuss the relevance of some of them as candidates for further studies in the search for new therapeutic targets in NE GI tumours.

## MATERIALS AND METHODS

### Cell lines

Human pancreatic carcinoid BON cells (Evers *et al*, 1991) were a generous gift from Professor Kjell Öberg, Department of Medical Sciences, Uppsala University Hospital, Uppsala, Sweden, and were grown in a 1 : 1 composition of Dulbecco's modified Eagle's medium (Gibco-BRL, Life Technologies, Paisley, Scotland) and RPMI 1640 medium (Gibco), containing 2 g l^−1^ glucose, 10 U ml^−1^ penicillin/streptomycin (Gibco) and 10% heat-inactivated foetal calf serum (FCS) (Biological Industries, Beit Haemek, Israel). The colon carcinoma LoVo cell line was obtained from the American Type Culture Collection (ATCC, Manassas, VA, USA), and grown in RPMI 1640 medium containing 10% FCS, 2 mM L-glutamine, 4.5 g l^−1^ glucose and 10 U ml^−1^ penicillin/streptomycin.

### Reagents

Heptadecapeptide-amidated gastrin and forskolin were obtained from Sigma (St Louis, MO, USA). Hepatocyte growth factor was a kind gift from Professor Magne Børset, Department of Cancer Research and Molecular Medicine, Norwegian University of Science and Technology, Trondheim, Norway. Recombinant EGF was purchased from Biomedical Technologies (Stoughton, MA, USA), and PACAP-38 from Bachem (Bobendorf, Switzerland).

### Proliferation

Proliferation rate was determined by measuring DNA synthesis using the Cell proliferation ELISA BrdU (5-bromo-2′-deoxyuridine) kit (Roche Applied Science, Mannheim, Germany). A total of 2 or 4 × 10^3^ BON cells were seeded out in 96-well microtitre plates in serum-containing medium. After 24 h, the cells were washed once with serum-free medium and then treated with various growth factors for 24 h. Incorporation of BrdU was measured as described previously (Hofsli *et al*, 2002).

### Radioimmunoassays (RIAs)

For detection of gastrin, BON cells were grown to confluence in six-well tissue culture plates and serum starved for 24 h in a volume of 1.2 ml, before the medium was collected, and cell lysates prepared by lysis in 400 *μ*l distilled water. Samples were kept frozen at −80°C before amidated gastrin was measured using an RIA method as described previously (Kleveland *et al*, 1985).

### Detection of c-Met, CCK2 and PAC_1_ receptor mRNA

Total RNA (2 *μ*g) from BON cells was reverse transcribed (42°C/60 min) in a volume of 20 *μ*l using *Mul*V reverse transcriptase (RT) (Roche Applied Science). A measure of 2 *μ*l of a 1 : 3 dilution of cDNA was then amplified in a 20 *μ*l polymerase chain reaction (PCR) mix (Roche Applied Science) containing a final concentration of 1.6 mM MgCl_2_, 500 nM of each primer, 1 U of AmpliTaq Gold and 300 *μ*M dNTP. PCR amplifications were run for 36 cycles with annealing temperature of 58°C (c-Met), 64°C (CCK2) and 56°C (PAC_1_) receptor, respectively. The following primers were used (-S designates sense and -AS antisense primer): c-Met-S – 5′-TGGGAATCTGCCTGCGAA-3′; c-Met-AS, 5′-CCAGAGGACGACGCCAAA-3′ (Børset *et al*, 1996); CCK2-S – 5′-AAC ACA AAC CAC AAC TGA-3′; CCK2-AS – 5′-AGG TCA AAC TAG GAG CAT-3′ (GenBank accession no. reference sequence; NM 176875); and PAC_1_-R-S – 5′-CCC TGA GCT CTT CCG AAT-3′; PAC_1_-R-AS – 5′-CCC ACA GGC ATC AAA GTA-3′ (NM 001118).

### Treatment of cells and isolation of RNA

For determination of growth factor-induced gene expression, BON cells were cultured in 75 cm^2^ culture flasks for 72 h until confluence was reached. After serum starvation for 24 h, cells were exposed to either gastrin (10 nM), HGF (100 ng ml^−1^), EGF (100 ng ml^−1^) or PACAP (10 nM) for 6 h. Untreated cells served as controls. RNA was isolated using the RNeasy Midi kit (Qiagen, Valencia, CA, USA), according to the manufacturer's instructions. RNA was examined for degradation by agarose gel electrophoresis with evaluation of the 18S and 28S ribosomal RNA bands and by use of Agilent 2100 Bioanalyzer (Agilent Technologies, Palo Alto, CA, USA). RNA concentration was assessed spectrophotometrically. The samples were kept frozen at −80°C until further processing.

### Microarray hybridisation

Arrays were manufactured by the Norwegian Microarray Consortium (www.mikromatrise.no/) using cDNA probes representing 2503 sequence-verified human genes (Research Genetics, Huntsville, AL, USA), including 1500 genes defined at that time in the NCI Oncochip selection. Of the 2503 genes, 2501 were printed in duplicates and two in quadruplicates. Additional information of cDNA clone preparation and printing are given in Nørsett *et al* (2004) and Yadetie *et al* (2003). Total RNA (1 *μ*g) from growth factor-treated and from untreated control cells (at time point zero) was reverse transcribed and labelled with Cy5- and Cy3-attached dendrimer, respectively, using the Genisphere 3DNA dendrimer kit (Genisphere, Montvale, NJ, USA) as described in the manufacturer's protocol and previously by us (Nørsett *et al*, 2004; Yadetie *et al*, 2003). Hybridised arrays were scanned separately at two wavelengths (532 and 633 nm).

### Microarray data analysis

Image analysis was carried out with the Microarray Suite software version 2.1 (Scanalytics, Fairfax, VA, USA). All subsequent statistical analyses were performed using the statistical package R, (R Development Core Team, 2004). Preprocessing consisted of automatic removal of weak spots and removal of systematic errors by normalisation. Spots with background-corrected spot intensity lower than the local background intensity in at least one channel were excluded from the analysis. Intensity-based normalisation was performed using locally weighted scatterplot smoothing (Yang *et al*, 2002).

Differentially regulated genes for each of the four treatments (gastrin, HGF, EGF and PACAP) were identified in a two-step procedure for each gene separately. First, a linear mixed effects model was fitted to the normalised log ratios of each gene, with the microarray slide as a random effect to take into account the correlation between multiple spots of each gene on the same slide. The nlme-software presented in Pinheiro and Bates (2000) was used to fit the model, and to produce a *P*-value for each gene based on a *t*-test in the fitted linear mixed effects model. In this first step, genes with *P*-values below 0.05 were selected as potentially interesting genes. It is, however, well known that gene-specific variances estimated from small sample sizes are unstable. To avoid that small estimated variances dominate genes with small estimated effects, a ratio cutoff of 0.8 and 1.25 was used on the selected genes.

To check the robustness of our analyses, we also calculated *P*-values based on a moderated *t*-test, as implemented in the Limma R package of Smyth (2004). The moderated *t*-test is based on empirical Bayes analysis, and is equivalent to shrinkage (or expansion) of the estimated sample variances towards a pooled estimate, resulting in more stable inference when the number of microarray experiments is small. A similar effect was achieved using the ratio cutoff in our two-step procedure. Duplicate spotted genes were averaged before the moderated *t*-tests were performed. The results to be presented are based on the two-step procedure, but in addition the maximum *P*- and *q*-values (Storey and Tibshirani, 2003) from the moderated *t*-tests on the selected gene sets are reported. The *q*-value of a gene can be interpreted as the minimum false discovery rate that can be attained when calling a given gene significantly differentially expressed. We have calculated a conservative version of the *q*-values, using 1 as the estimated proportion of genes truly not differentially expressed.

### Quantitative RT–PCR

Verification of the microarray data was performed by quantitative RT–PCR analysis using the Smart Cycler (Cepheid, Sunnyvale, CA, USA). First, total RNA in a final concentration of 0.1 *μ*g *μ*l^−1^ was reverse transcribed (48°C/60 min) using 1 U *μ*l^−1^ of Euroscript RT (Reverse Transcription Core Kit, Eurogentec, Seraing, Belgium). Undiluted cDNA (2 *μ*l) were then amplified for 30–40 cycles in a 25 *μ*l PCR mix (qPCR™ Core Kit for Sybr™ Green I-No ROX) (Eurogentec) containing a final concentration of 2.5 mM MgCl_2_ and 300 nM of each primer. Each cycle consisted of denaturing for 15 s at 95°C, annealing for 15 s at 55°C and polymerisation for 30 s at 72°. The *C*_t_ value, defined as the number of cycles required to produce a detectable product above background fluorescence, was measured for each sample, and arbitrary units were calculated using a standard curve, which was run for each individual PCR analysis. The standard curves consisted of serial dilutions of cDNA of a sample or a control containing the highest amount of the specific gene analysed. A negative control without the cDNA template was run with every PCR assay, and contamination by genomic DNA was ruled out by performing PCR analysis on template where RT had been omitted in the RT reactions. Glyceraldehyde-3-phosphate dehydrogenase RT–PCR were run in parallel as control to monitor RNA integrity and to be used for normalisation. Specificity of each primer pair was confirmed by melting curve analysis and agarose gel electrophoresis. Table 1 shows the primer sequences and expected sizes of the PCR products.

## RESULTS

### Gastrin-induced proliferation

**Figure 1:**
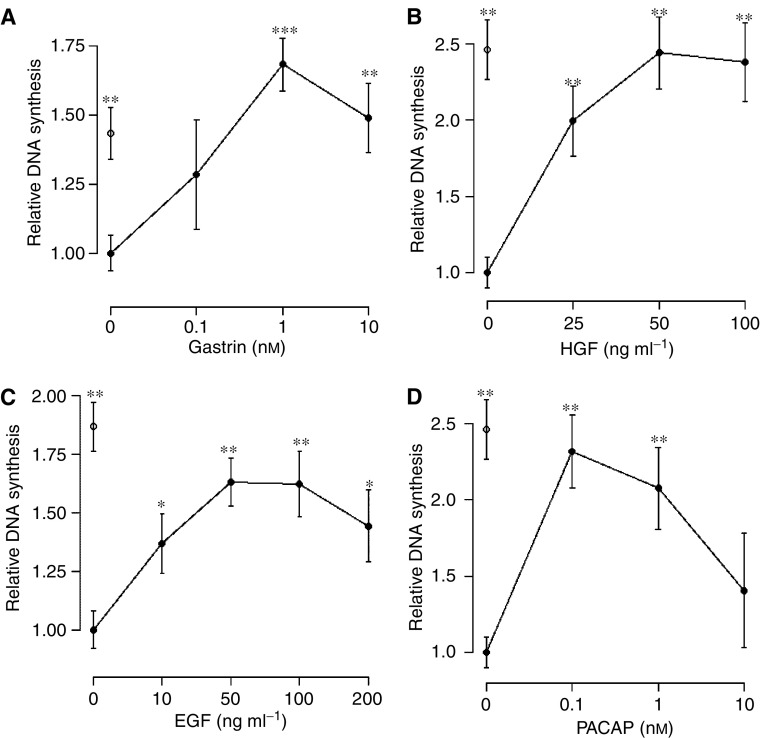
Growth factor-induced proliferation of BON cells. BON cells were stimulated for 24 h with gastrin (**A**), HGF (**B**), EGF (**C**) or PACAP (**D**), with quadriplicate parallels per condition, and proliferation measured as BrdU incorporation. Results are shown as the mean value±s.e.m. of one representative experiment, and are expressed as relative values compared to untreated cells (control). Similar results were obtained in at least three other experiments. ^*^P⩽0.01. ^**^P⩽0.001, ^***^P⩽0.0001. ○, proliferative effect of 10% FCS. The statistical test used is an unpaired *t*-test.

Since gastrin appears to be an important growth factor of various tumour cell types (Dockray *et al*, 2001, 2005; Richards *et al*, 2004), and since some gastric carcinoids have been shown to express the CCK2 receptor (Smith *et al*, 1998), we examined whether gastrin could affect growth of carcinoid tumour cells as well. As shown in Figure 1A, gastrin induced proliferation of BON cells in a dose-dependent manner. A maximum 1.7-fold increase was seen, which was comparable to that induced by FCS (1.4). To explore a possible role of gastrin as an autocrine growth factor of BON cells, we measured the gastrin protein levels in cell lysates and in conditioned medium. Our finding that BON cells do not produce gastrin themselves (detection limit in our assay is 5 pg ml^−1^) is in agreement with immunohistochemical findings of Evers *et al* (1991).

**Figure 2:**
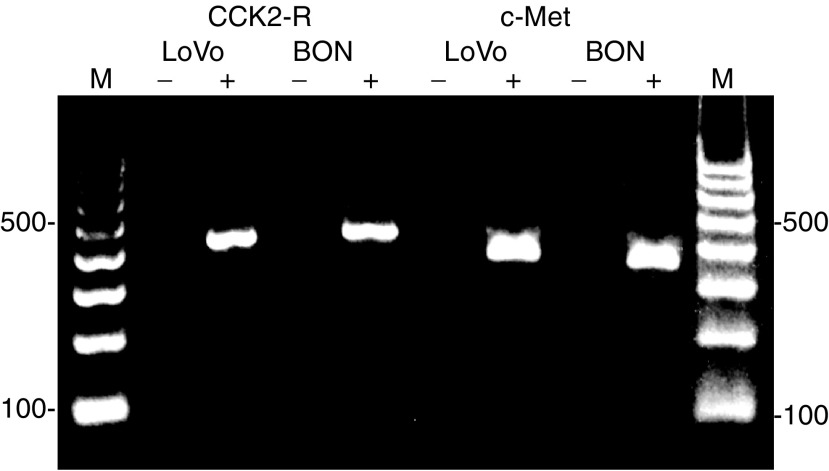
Expression of CCK2 receptor and HGF receptor (c-Met) in BON cells. RT–PCR analysis of total RNA was performed with (+) or without (−) RT to rule out contamination of genomic DNA. PCR products were visualised in ethidium bromide-stained 1.2% agarose gels, and showed the expected sizes of 398 bp (CCK2 receptor) and 395 bp (c-Met), respectively. LoVo cells were used as a positive control for CCK2 receptor (Watson *et al*, 1998) and c-Met (Nabeshima *et al*, 1998). The results shown are representative of three independent experiments. M: 100 bp DNA ladder molecular weight marker.

Since there have been conflicting results as to the question whether BON cells express the CCK2 receptor (Evers *et al*, 1991; Kim *et al*, 1997), we analysed for its mRNA by RT–PCR analysis. The results clearly demonstrate that BON cells express CCK2 receptor mRNA (Figure 2). Although this finding does not necessarily mean that the CCK2 receptor protein is being expressed in BON cells, it indicates that the observed growth-stimulatory effect of gastrin is mediated via this receptor. Together, our findings support the view that gastrin may act as a regulator of carcinoid tumour growth (Smith *et al*, 1998; Behe and Behr, 2002).

### HGF-, EGF- and PACAP-induced proliferation

In order to further explore growth regulation of human NE GI tumour cells, we tested a panel of other factors known to affect cell growth of other tumour cell types. We here report the novel findings that HGF, EGF and PACAP all stimulate proliferation of carcinoid BON cells (Figure 1B–D). A maximum 2.4-fold increase in proliferation was seen after stimulation with 50 ng ml^−1^ of HGF. By RT–PCR analysis, BON cells were shown to express mRNA of the HGF receptor (c-Met) (Figure 2), indicating a possible role of HGF in controlling growth of carcinoids. This is perhaps not surprising, as an aberrant c-Met signalling, either caused by overexpression/mutation of c-Met or by coexpression of c-Met and HGF, has been found in a variety of other malignancies (Maulik *et al*, 2002). Only two studies have so far investigated c-Met expression in NE GI tumours. Wulbrand *et al* (1998) found the c-Met mRNA expression frequencies to vary considerably, with the highest found in gastrinomas (33%) and the lowest (11%) in small intestine carcinoid tumours. In a gastrinoma study (Peghini *et al*, 2002), universal expression of c-Met mRNA was demonstrated in all 22 tumours investigated. Compared to normal pancreas, only a minority of the tumours (14%) showed slight overexpression of c-Met. Unlike findings in other tumour types (Maulik *et al*, 2002), c-Met overexpression in these gastrinomas were not correlated with a more aggressive tumour behaviour. In conclusion, the present study indicates that the BON cell line could serve as a valuable experimental model to study molecular mechanisms involved in HGF-mediated responses in NE tumour cells.

Our finding that EGF stimulates growth of BON cells (Figure 1C) was somewhat surprising, since others did not find this effect in BON (Townsend *et al*, 1993). A maximum 1.6-fold increase was seen using either 50 or 100 ng ml^−1^ EGF. The discrepancy of results may relate to the fact that we used a more sensitive method for measurement of proliferation, or to phenothypic changes that may have occurred during cultivating of cells for years *in vitro*. Western blot analysis revealed a constitutive expression of phosphorylated (activated) EGF receptor in BON cells (data not shown), indicative of an aberrant EGF signalling in these cells. Recently, EGFR expression has in fact been demonstrated in BON cells (Höpfner *et al*, 2003), but BON cells were not found to express the EGFR mutation EGFRvIII, which is often observed in non-NE tumours.

Also, PACAP was found to enhance DNA synthesis in BON cells (Figure 1D). Both the concentration of 0.1 and 1 nM PACAP increased the proliferation by a factor of 2.2. Only a weak increase was observed with 10 nM PACAP. By RT–PCR analysis, BON cells were found to express mRNA of the PACAP selective receptor PAC_1_, but not VIP/PACAP receptors (data not shown). This is in agreement with earlier findings showing that tumours located in the NE system appear to express predominantly the PAC_1_ receptor (Reubi *et al*, 2000). Furthermore, it is consistent with findings in this study showing that PACAP in the range of 0.1–10 nM induces proliferation of BON cells, and with those in a previous study from our group showing that PACAP at the same concentrations induces histamine release from ECL cells known to only possess PAC_1_ and not VIP/PACAP receptors (Sandvik *et al*, 2001). No previous studies have investigated the expression of PACAP and its receptors in carcinoids. Our assumption that PACAP could act as an important growth regulator of NE GI tumours is supported by the fact that PACAP has been found to modulate rat gastric ECL cell proliferation, a cell type which during malignant transformation gives origin to gastric carcinoid tumours (Läuffer *et al*, 1999).

### Gastrin- and HGF-regulated genes

Having established the proliferative responses to gastrin, HGF, EGF and PACAP in BON cells, we performed transcript profiling by cDNA microarray analysis in an effort to improve insight into molecular mechanisms underlying these responses. A pilot study where gene expression was analysed in BON cells treated with gastrin for 2, 6, 16 or 24 h demonstrated that the number of differentially expressed genes was highest at 6 h (data not shown). We therefore used this time point for further microarray analysis. We particularly focused on gastrin- and HGF-induced gene expression for three reasons. First, there are reasons to believe that gastrin plays a role as a regulator of carcinoid tumour growth *in vivo*. Second, the novel finding that also HGF affects growth of carcinoid tumour cells is interesting considering the central role HGF plays as a growth regulator of other tumour cell types. Third, gastrin and HGF represent growth factors acting through two different main types of receptors: G-protein-coupled and tyrosin kinase receptors, respectively.

Among the 2503 genes analysed, inference was performed for 2050 genes, where the number of observations was at least 3. Using our two-step statistical procedure to identify differentially expressed genes, we identified 95 genes to be regulated by gastrin, of which 54 were up- and 41 were downregulated (Supplementary Data). Furthermore, we identified 101 genes to be regulated by HGF, of which 47 were up- and 54 downregulated (Supplementary Data). These results were compared to the use of the moderated *t*-tests using the Limma R package of Smyth (2004). The maximum Limma *P*-value for the 95 potentially gastrin-regulated genes was found to be 0.01 and the maximum *q*-value to be 0.17. For the 101 potentially HGF-regulated genes, the maximum Limma *P*-value was 0.01 and the maximum *q*-value 0.12. We have previously shown that the labelling method used in the present study results in compressed microarray ratios, but quantitative RT–PCR validation experiments performed here (Table 3) and previously (Yadetie *et al*, 2003; Nørsett *et al*, 2004) have been able to confirm the microarray data.

Based on information from the literature and from the LocusLink and SwissProt databases, the genes were grouped according to cellular processes in which they are likely to be involved. Since we were interested in processes that are likely to play a role in the context of cancer, we focused on cell proliferation, apoptosis, cell adhesion, cell motility and oncogenesis. Even though many genes may be involved in more than one process, each of the genes was grouped into only one category; this regarded to be most relevant for the response studied here (Table 2). Within these categories, we identified 27 genes that are regulated by both gastrin and HGF (Table 2a), 30 that are only regulated by gastrin (Table 2b), and finally, 25 that are only regulated by HGF (Table 2c). The remaining 38 gastrin- and 49 HGF-responsive genes were regarded to participate in other cellular processes, or to have unknown functions (all differentially expressed genes are shown in Supplementary Data). Of the 95 and 101 gastrin- and HGF-responsive genes, similar proportions were involved in cell proliferation (23–24%), cell motility/adhesion (15–16%) and in apoptosis (4%). It was therefore interesting to note that for the process oncogenesis, the proportion of gastrin-responsive genes was twice as high (16%) as the proportion of HGF-responsive genes (8%). This may indicate that the oncogenic potential of gastrin in carcinoid cells is at least as high as that of HGF.

### Genes regulated by all growth factors

In order to identify common growth factor-responsive genes, which may represent candidate genes of tumour growth regulation in general, we compared gene expression induced by gastrin, HGF, EGF and PACAP. In all, 12 genes were found to be significantly regulated (P<0.05 from the *t*-test based on the linear mixed effects model in the first step of our two-step procedure) by all the four growth factors (Table 3), indicating that they may be universally regulated during NE GI tumour cell proliferation. However, it is to be noted that the concentration of PACAP (10 nM) used in the gene expression analysis only induced a weak proliferative response in BON cells (Figure 1D). The common genes identified participate in various cellular processes, as four of them encode proteins known to be mediators of proliferation (*EDN1*, *EGR3*, *ATF4* (activating transcription factor 4), *IL2RB*), three encode proteins involved in adhesion (*CCR1*, *CDH11* (cadherin-11), *LAMA5* (laminin, alpha 5)), two in metabolism (*PCCA*, *PFKM*), one in RNA splicing (*PPIG*), and finally, two are involved in oncogenesis (*CIN85*, *PIM1*).

### Validation by real-time quantitative RT–PCR

It is well known that data from microarray analysis need to be interpreted cautiously (Kothapalli *et al*, 2002). To validate the microarray results in this study, we performed real-time quantitative fluorescent RT–PCR analysis of seven selected genes using the same RNA samples from gastrin- and HGF-treated cells as those used in the microarray analysis. Concordant results were found for all the four upregulated genes tested (*ATF4*, *LAMR1* (laminin receptor 1), *BTG1*, *RRM2*) (Table 4), while one (*IGFBP1*) out of three downregulated genes tested (*EPS15*, *IGFBP1*, *PIM1*) was confirmed to be differentially expressed (Table 4). The serine/threonine kinase *PIM1* proto-oncogene was, by RT–PCR analysis, shown to be slightly upregulated by gastrin, and markedly upregulated by HGF. In prostate cancer, the expression of the *PIM1* protein has been shown to correlate with clinical outcome (Dhanasekaran *et al*, 2001), and the novel detection of this proto-oncogene in carcinoid tumour cells warrants further studies to explore its role as a potential prognostic marker also in NE GI tumours.

Our confirmatory rate of 71% underlines the need to verify microarray data by other methods, but our verification results seem to be in accordance with that of others (Kothapalli *et al*, 2002) and that previously shown by us (Nørsett *et al*, 2004; Yadetie *et al*, 2003).

## DISCUSSION

Growth factor-induced signalling impacts on many aspects of tumour biology. The results of this study contribute to an increased knowledge of molecular events underlying growth factor-induced proliferation of human NE GI tumour cells, by identifying a number of novel growth-responsive genes. Our study also sheds new lights into the biology of NE GI tumours, and provides candidate genes in the search for new therapeutic targets.

To the best of our knowledge, only a few of the growth factor-responsive genes identified in this study have earlier been shown to be regulated by the respective factors. However, the demonstration of differential expression of already known growth factor-responsive genes confirms the reliability of our results. In a study investigating the transcriptional response to HGF in mouse embryo liver cells, HGF was found to regulate a number of extracellular matrix proteins (Medico *et al*, 2001). Similar to the findings in that study, we confirmed the upregulation of the gene *COL1A2* (collagen, type I, alpha), and the downregulation of *LAMA5* by HGF. Furthermore, gastrin was shown to upregulate the expression of interleukin-8 (IL-8), which has earlier been identified as a gastrin-regulated gene in rat gastric epithelial cells (Hiraoka *et al*, 2001). IL-8 has been shown to contribute to human cancer progression through its mitogenic, angiogenic and migratory action (Xie, 2001). In accordance with a recently published study performed using rat pheochromocytoma PC12 cells (Grumolato *et al*, 2003), we identify *LAMR1* as a PACAP-responsive gene. Finally, tissue inhibitor of metalloproteinase 3 (TIMP3) is recognised as an HGF-responsive gene, as previously shown by Castagnino *et al* (1998).

Annotations of the genes reveal that the majority of the novel gastrin- and HGF-responsive genes seem to participate in processes relevant in the context of tumour biology. However, it has to be noted that cancer-related genes were well represented on our arrays. In addition to IL-8, several other genes encoding proteins involved in *cell proliferation* were found to be regulated by gastrin and/or HGF. Gastrin was shown to upregulate the mRNA expression of the small M2 subunit of ribonucleotide reductase (*RRM2*), a rate-limiting enzyme in DNA synthesis and repair (Zhou and Yen, 2001). Several nonspecific inhibitors of ribonucleotide reductase are in fact already in clinical use as chemotherapeutic agents, and more recently, an antisense agent targeting RRM2 has shown potent antitumour activity against a variety of tumours (Lee *et al*, 2003). As far as we know, our study is the first one to demonstrate that polypeptide growth factors may affect the expression of RRM2, as also PACAP was shown to induce an upregulation.

Gastrin, HGF and PACAP were all shown to upregulate the expression of the highly conserved *LAMR1* gene that plays a role in *cell adhesion*. This is interesting as there seems to be a correlation between the upregulation of LAMR1 in cancer cells, and their invasive and metastatic phenotype (Menard *et al*, 1998). Furthermore, gastrin was shown to downregulate the expression of spleen tyrosine kinase (*SYK*) gene. Reduced expression of this gene has been shown to be associated with distant metastasis and poor prognosis in breast cancer (Toyama *et al*, 2003), indicating that it may be a potential tumour suppressor and antimetastasis gene. Thus, downregulation of *SYK* may contribute to the assumed tumorigenic potential of gastrin. As elevated serum levels of the tumour-associated glycoprotein lectin, galactoside-binding, soluble, 3 binding protein (*LGALS3BP*) (Table 2a) are often observed in cancer patients, and have been proven to be of prognostic value (Marchetti *et al*, 2002), the findings that both gastrin and HGF upregulate the expression of LGALS3BP justify studies exploring LGALS3BP expression in carcinoid tumour patients as well.

Gastrin and HGF affected the expression of several genes encoding proteins thought to be involved in *apoptosis* (Table 2a–c). As mentioned above, TIMP3 has earlier been recognised as an HGF-responsive gene, and here we demonstrate a marked downregulation of this proapoptotic gene after treatment with HGF. This is interesting as we know that loss of TIMP3 expression may enhance the invasive potential of certain tumours (Castagnino *et al*, 1998). Moreover, gastrin was shown to downregulate the expression of lymphotoxin beta receptor (*LTBR*). Activation of this receptor has been shown to induce apoptosis in some tumour cells (Browning *et al*, 1996), and thus a downregulation of its expression probably undermines its apoptotic effect.

It is noted that a considerably higher percentage of gastrin- than HGF-regulated genes were found to encode proteins considered to be involved in *oncogenesis*. Aberrant HGF/c-Met signalling has been well documented in a variety of human cancers (Maulik *et al*, 2002), and our results strengthen the view that also the gastrin signalling pathway could be involved in oncogenesis (Waldum *et al*, 1995; Dockray *et al*, 2001). An abberant expression of the homeo box A1 (*HOXA1*) gene is assumed to be the mechanism by which autocrine stimulation with human growth factor may result in oncogenic transformation, and it has recently been shown that forced expression of this gene in human mammary epithelial cells is sufficient for transformation of the cells (Zhang *et al*, 2003). Our finding that gastrin upregulates the expression of the *HOXA1* gene indicates that this may be one mechanism by which gastrin could play a role in oncogenesis. Another mechanism could be through an upregulation of the mRNA of S100 calcium-binding protein A3 (*S100A3*), as this gene has been proposed to play a role in NE tumorigenesis (Stålberg *et al*, 2001). Additional support to such a view is the finding that gastrin downregulates the expression of the chromosome condensation 1-like (*CHC1L*) gene. This gene has recently been proposed to be a candidate gene for prostate carcinogenesis (Latil *et al*, 2003).

Crosstalks between various growth factor systems are a well-known phenomenon, and underline the complexity of tumour growth control. Gastrin has previously been shown to enhance the expression of both HGF (Konturek *et al*, 2003) and heparin-binding EGF-like growth factor (Dockray *et al*, 2001). In the present study, links to the IGF system were observed. This is of particular interest since IGF-1 is known to act as an autocrine growth factor of BON cells (von Wichert *et al*, 2000). Gastrin was shown to downregulate the expression of the *IGFBP-1* (Tables 2b and 4). IGFBP-1 is known to neutralise the actions of IGF-1, but in addition, it exerts independent actions as it inhibits breast cancer cell motility and growth by itself (Zhang and Yee, 2002). In fact, in breast cancer several strategies are currently being exploited using the IGF system as a biological target (Yee, 2002). Thus, a downregulation of IGFBP1 by gastrin may be one mechanism whereby gastrin promotes tumour growth and invasion. Another link to the IGF system was also noted, as HGF was shown to upregulate the expression of the IGF receptor 2 (*IGFR2*). Our data also indicate connections to other growth factor systems, as gastrin was shown to upregulate the expression of acidic fibroblast growth factor 1 (*FGF1)*, while both gastrin and HGF were shown to decrease the expression of EGF receptor pathway substrate 15 (*EPS15*). HGF also increased the expression of PDGF receptor alpha polypeptide (*PDGFRA*), while PACAP was shown to upregulate the expression of the HGF-receptor c-Met.

PACAP was shown to induce a rather strong upregulation of IL-8. This is similar to gastrin, which also acts through a G-protein-coupled receptor. In contrast, no regulation was seen after treatment with the tyrosine kinase-acting ligands HGF and EGF. A similar pattern was observed with regard to RRM2 expression, as the tyrosine kinase receptor-activating ligands HGF and EGF did not affect the expression of RRM2, while gastrin and PACAP upregulated RRM2 RNA levels.

One objective of this study was to identify genes that were regulated by all four growth factors, since such genes could possibly be regarded as universally regulated during tumour cell proliferation, and thus assumed to be of particular interest in the search for new drug targets. In all, 12 genes fulfilled the criteria, indicating that these genes may play a central role in NE GI tumour cell proliferation. Gastrin, HGF, EGF and PACAP were all shown to increase strongly the mRNA of *ATF4*, a member of the ATF/CREB family of mammalian transcription factors found to be expressed in all tissues examined so far (Hai and Hartman, 2002). Heregulin, a combinatorial ligand for EGFR 3 and 4, has earlier been found to upregulate ATF4 mRNA as well as ATF4 protein in human cancer cells (Talukder *et al*, 2000). Together, this indicates that ATF4 could play a central role in the mechanisms of tumour growth regulation in general.

Another interesting feature observed with all four growth factors was marked downregulation of the beta chain of the interleukin 2 receptor (*IL2RB*). IL2 is known to inhibit proliferation and growth of various cancer cells (Casana *et al*, 2002), and thus a downregulation of a subunit of its receptor may be one additional mechanism whereby growth factors in general promote growth. All four growth factors were shown to downregulate the expression of *CDH11*. This has previously been shown to be the case also after treatment with transforming growth factor-*α* (Zhou and Skalli, 2000). As CDH11-overexpressing osteosarcoma cells have been found to exhibit a marked reduction in their ability to form pulmonary metastasis (Kashima *et al*, 2003), a downregulation of CDH11 may be one mechanism by which growth factors contribute to tumour progression. Finally, all growth factors were shown to downregulate the expression of LAMA5, which is interesting as a reduced level of LAMA5 has been shown to be associated with an increased tendency to develop lymph node metastasis in non-small-cell lung cancer (Akashi *et al*, 2001).

Our data showing that all four growth factors analysed may induce the expression of genes known to impact on many aspects of cancer indicate that these factors may play an important role in the establishment, growth and metastases of NE GI tumours, and that targeting their receptors, signalling pathways or secondary gene expression may be a possible way to stop or delay growth of such tumours. As mentioned above, targeting the EGF system has already become an established treatment modality in some types of tumours (Sridhar *et al*, 2003; Ranson, 2004), and has been shown to inhibit growth of NE GI tumour cells (Höpfner *et al*, 2003). Similarly, the pivotal role that HGF seems to play in cancer has led to the preclinical development of many types of targeting drugs, including antagonists, which compete with binding to the receptor, antibodies, which block the receptor, small-compound tyrosin kinase inhibitors, or other strategies that target downstream signals or secondary gene expression (Jiang *et al*, 2005).

In light of the fact that tumour growth *in vivo* is most often influenced by many different growth factors, either in a paracrine, autocrine or systemic manner, our identification of genes that are regulated by more than one factor in NE GI tumour cells may be of particular interest with regard to intervention of NE tumour disease. In addition, the demonstration in this paper of crosstalks between various growth factor systems strengthens the view that it probably will be more efficient to target more than one growth systems, as more recently has been shown in NE medullary thyroid cancer preclinical models (Ezzat *et al*, 2005). Our identification of ATF4 as a common inducible growth factor gene may indicate that silencing of this gene, for example, by use of siRNAs or antisense technology, may be an interesting approach to further study. Furthermore, the demonstration that gastrin and PACAP, similar to tumour microenvironment factors such as hypoxia, acidosis and nitric oxide (Xie, 2001), regulate IL-8 expression underline the need to further investigate the use of IL-8-neutralising antibodies, IL-8 receptor blockers or IL-8 gene silencing approaches in the treatment of NE GI tumours. Moreover, LAMR1, also known as ribosomal protein SA, turns out to be an interesting candidate for further studies as both gastrin and HGF were shown to upregulate its expression. To the best of our knowledge, our study is the first to report LAMR1 expression in NE tumour cells.

In conclusion, by identifying a number of growth factor-responsive genes in human NE GI tumour cells, we have shed new light into the molecular mechanisms involved in growth control of these cells. Such knowledge is essential in order to design studies aimed to evaluate functional aspects of candidate genes. Although caution is advisable in the interpretation of microarray expression data, we believe that our results provide suggestions for further studies in the effort to identify new therapeutic targets as well as new markers of NE GI tumours.

## Figures and Tables

**Table 1:** Primer sequences of selected genes for confirmation studies

**Gene**		**PCR products (bp)**
*EPS15*	S-5′-CTG TCC CAA ACC CTT TAC A-3′	130
	AS-5′ AGA ATC CCA TCC ATA TAT CCA-3′	
		
*IGFBPI*	S-5′-GGA GGA CGG TTA ACT TGT AT-3′	130
	AS-5′-AAC AAC ATA TCC AGG TAC ATT-3′	
		
*PIM1*	S-5′-GGA GAC CCC AGA TAG GAC-3′	125
	AS-5′-GGA GAT AGG AAG CCA GAC TAC-3′	
		
*RRM2*	S-5′-AGC AAG CGA TGG CAT AG-3′	110
	AS-5′-GTA TGT TTT CCA TGG CAA TTT-3′	
		
*ATF4*	S-5′-GGA GAC CCC AGA TAG GAC TC-3′	110
	AS-5′-GAC TAC ACT GCT TAC GTT GCC-3′	
		
*LAMR1*	S-5′-TGA AAT TCC TCC TTG GTC ACT-3′	120
	AS-5′-CAT TTC CCG TGA ACA CCC-3′	
		
*BTG*	S-5′-CAA CTT TGC CAC AAT CAA TA-3′	141
	AS-5′-CTT TCC TTG TAC TTA ATT GGG-3′	
		
*GAPDH*	S-5′-TCT GAC TTC AAC AGC GAC ACC-3′	118
	AS-5′-TGT TGC TGT AGC CAA ATT CGT-3′	

PCR=polymerase chain reaction; S=sense; AS=antisense.

**Table 2:** Genes significantly^a^ regulated by (a) both gastrin and HGF, (b) gastrin and not by HGF and (c) HGF and not by gastrin

			**Ratio^c^**	
**Gene symbol**	**Gene name**	**Accession^b^**	**Gastrin**	**HGF**
*(a) Both gastrin and HGF*				
Cell proliferation				
ATF4	Activating transcription factor 4	AA600217	1.63	1.46
CCNI	Cyclin I	AA434408	1.52	1.42
ANXA11	Annexin A11	AA465051	1.47	1.27
MCM3	Minichromosome maintenance deficient 3	AA455786	1.35	1.38
NP	Nucleoside phosphorylase	AA430382	1.29	1.30
STK12	Serine/threonine kinase 12	AA071486	1.25	1.28
IL2RB	Interleukin 2 receptor, beta	AA057156	0.48	0.46
GNRH1	Gonadotropin-releasing hormone 1	AA043996	0.55	0.57
EPS15	EGF receptor pathway substrate 15	AA490223	0.66	0.63
LOC51177	CK2-interacting protein 1	AA490216	0.76	0.75
SCYA16	Small inducible cytokine subfamily A (Cys–Cys), member 16	T58775	0.77	0.70
				
Apoptosis				
BAK1	BCL2 antagonist/killer 1	H52673	1.26	1.29
HCLS1	Hematopoietic cell-specific Lyn substrate 1	AA424575	0.77	0.77
				
Cell adhesion/motility				
LGALS3BP	Lectin, galactoside-binding, soluble, 3 binding protein	AA485353	1.54	1.45
MAP1B	Microtubule-associated protein 1B	AA219045	1.54	1.33
LAMR1	Laminin receptor 1	AA629897	1.49	1.41
MIG	Monokine induced by gamma interferon	AA131406	0.53	0.66
CCR1	Chemokine (C–C) motif receptor 1	AA036881	0.63	0.60
CDH11	Cadherin 11	AA136983	0.66	0.65
LAMA5	Laminin, alpha 5	AA459519	0.77	0.72
ITGAV	Integrin, alpha V	AA029934	0.77	0.78
				
Oncogenesis				
MLF2	Myeloid leukemia factor 2	AA480835	1.30	1.40
CIN85	c-Cbl-interacting protein	AA989257	1.31	1.26
LMO2	LIM domain only 2	AA464644	1.29	1.31
GSTT1	Gluatathione *S*-transferase theta 1	H99813	1.25	1.35
PIM1	Pim-1 oncogene	AA453663	0.75	0.50
RAB18	RAB18, member RAS oncogene family	AA156821	0.79	0.75
				
**Gene symbol**	**Gene name**	**Accession^b^**	**Ratio^c^**	
*(b) Gastrin and not by HGF*				
Cell proliferation				
RRM2	Ribonucleotide reductase M2 polypeptide	AA187351	1.59	
BTG1	B-cell translocation gene 1	N70463	1.57	
IL-8	Interleukin 8	AA102526	1.46	
EGR3	Early growth response 3	R39111	1.45	
ERCC5	Excision repair crosscomplementing rodent repair deficiency, complementation group 5	N62586	1.44	
MCM3	Minichromosome maintenance deficient 3	AA455786	1.35	
FGF1	Fibroblast growth factor 1 (acidic)	AA015793	1.30	
ETR101	Immediate-early protein	AA496359	1.27	
RAD23A	RAD23 (*C. cervisiae*) homolog A	AA476274	1.26	
CAMK2D	Calcium/calmodulin-dependent protein kinase (camkinase) II delta	AA283023	1.26	
LIG3	Ligase III, DNA, ATP dependent	AA149292	0.61	
HDAC2	Histone deacetylase 2	AA127093	0.72	
				
Apoptosis				
PPARG	Peroxisome proliferative-activated receptor, gamma	A088517	1.3	
LTBR	Lymphotoxin beta receptor	AA454646	0.67	
				
Cell adhesion/motility				
RCN2	Reticulocalbin 2	AA598676	1.36	
SERPINA5	Serine (or cysteine) proteinase inhibitor, member 5	AA858026	1.28	
CDH6	Cadherin 6	AA421819	1.25	
SYK	Spleen tyrosine kinase	AA598572	0.63	
IGFBP1	Insulin-like growth factor binding protein 1	AA233079	0.66	
SCYA2	Small inducible cytokine A2	AA425102	0.71	
MMP7	Matrix metalloproteinase 7	AA031513	0.78	
				
Oncogenesis				
HOXA1	Homeo box A1	AA173290	1.33	
S100A3	S100 calcium-binding protein A3	AA055242	1.29	
EWSR1	Ewing sarcoma break point region 1	AA464184	1.29	
UBE3A	Ubiquitin protein ligase E3A	N94099	1.29	
SIAT4C	Sialyltransferase 4C	AA453898	1.25	
CHC1L	Chromosome condensation 1-like	AA495766	0.66	
LBC	Lymphoid blast crisis oncogene	AA156936	0.70	
MEL	Mel transforming oncogene	AA064715	0.77	
OS-9	Amplified in osteosarcoma	AA03336	0.77	
				
*(c) HGF and not by gastrin*				
Cell proliferation				
ELK4	ETS-domain protein (SRF accessory protein 1)	H61758	1.55	
MAT2A	Methionine adenosyltransferase II, alpha	T59286	1.53	
MAPK4	Mitogen-activated protein kinase 4	AA401035	1.40	
IGF2R	Insulin-like growth factor 2 receptor	T62547	1.32	
PDGFRA	PDGF receptor alpha polypeptide	H23235	1.27	
CDC6	CDC6 homolog	H59203	1.26	
GSK3B	Glycogen synthase kinase 3 beta	R93911	1.26	
BDNF	Brain-derived neurotrophic factor	AA262988	0.66	
TNFRSF11B	TNF receptor superfamily, member 11	AA194983	0.67	
JUND	Jun D proto-oncogene	AA418670	0.73	
TSC22	TGF beta-stim protein TSC-22	AA664389	0.75	
STATI2	STAT induced STAT inhibitor 2	AA137031	0.78	
TOB1	Transducer of ERBB2,1	AA490213	0.79	
				
Apoptosis				
TIMP3	Tissue inhibitor of metalloproteinase 3	AA099153	0.55	
BIRC1	Baculoviral IAP repeat-containing 1	AA621150	0.80	
				
Cell adhesion/motility				
ACTG2	Actin, gamma 2, smooth muscle, enteric	T60048	1.52	
COL1A2	Collagen, type I, alpha 2	AA490172	1.35	
MYPT2	Myosin phosphatase, target subunit 2	AA463926	1.36	
DSP	Desmoplakin (DPI, DPII)	H90899	1.30	
PCDH1	Protocadherin 1 (cadherin-like 1)	AA443557	1.27	
CD164	CD164 antigen, sialomucin	AA598561	0.55	
SDC4	Syndecan-4	AA148736	0.72	
CD58	CD58 antigen	AA136359	0.75	
				
Oncogenesis				
WT1	Wilms tumor 1	AA130187	0.76	
KIAA0203	KIAA0203 gene product	AA047435	0.76	

HGF=hepatocyte growth factor.

a Significantly (*P*⩽0.05) differentially expressed genes with a microarray ratio <0.8 or >1.25 using the two-step procedure outlined in the Materials and Methods section.

b GenBank accession number. Listed are genes with functions relevant in cancer biology. The remaining genes with other or unknown functions are listed in Supplementary Data. The gastrin data are based on samples from two biological experiments, of which one sample was hybridised once, and the other twice. The HGF data are based on samples from two biological experiments, which both were hybridised twice.

c Microarray ratio (treated/untreated cells).

**Table 3:** Genes significantly^a^ regulated by all growth factors

**Gene symbol**	**Gene name**	**Accession^b^**
*Upregulated*		
EDN1	Endothelin 1	H11003
PCCA	Propionyl coenzyme A carboxylase, alpha polypeptide	AA608575
PPIG	Peptidyl-prolyl isomerase G	AA458502
CIN85	c-Cbl-interacting protein	AA989257
EGR3	Early growth response 3	R39111
ATF4	Activating transcription factor 4	AA600217
		
*Downregulated*		
IL2RB	Interleukin 2 receptor, beta	AA057156
CCR1	Chemokine (C–C motif) receptor 1	AA036881
CDH11	Cadherin 11, type 2, OB-cadherin (osteoblast)	AA136983
PFKM	Phosphofructokinase, muscle	AA099169
PIM1	Pim-1 oncogene	AA453663
LAMA5	Laminin, alpha 5	AA459519

a Genes significantly (*P*<0.05) down- or upregulated by gastrin, HGF, EGF and PACAP using the first step of the two-step procedure outlined in the Materials and Methods section.

b GenBank accession number. The gastrin data are based on samples from two biological experiments, of which one sample was hybridised once, and the other twice. The HGF data are based on samples from two biological experiments, which both were hybridised twice.

**Table 4:** Verification of microarray data by quantitative RT–PCR analysis

**Gene**	**Treatment**	**PCR ratio^a^**	**Microarray ratio^b^**
*ATF4*	Gastrin	2.1	1.6
	HGF	1.5	1.5
			
*BTG1*	Gastrin	1.2	1.5
	HGF	1.2	1.2
			
*RRM2*	Gastrin	1.2	1.7
	HGF	1.0	1.2
			
*LAMR1*	Gastrin	1.3	1.6
	HGF	1.1	1.3
			
*IGFBPI*	Gastrin	0.9	0.7
	HGF	0.8	0.8
			
*EPS15*	Gastrin	1.0	0.7
	HGF	NA	0.6
			
*PIM1*	Gastrin	1.8	0.8
	HGF	1.1	0.6

RT–PCR=reverse transcriptase–polymerase chain reaction; HGF=hepatocyte growth factor; NA; not analysed.

a RNA transcript level in arbitrary units was standardised by the level of GAPDH, and expressed as fold change in treated relative to untreated cells. RT–PCR analysis was performed with the same total RNA as used in the microarray experiments. PCR ratios calculated from analysis of one biological sample with either gastrin or HGF are shown.

b Corresponding microarray ratio for the same biological sample.
