# Supplementary material for: Identification of novel growth factor-responsive genes in neuroendocrine gastrointestinal tumour cells
Source: Br J Cancer. 2005 Apr 20;92(8):1506–16. doi: 10.1038/sj.bjc.6602535 (PMC2361991; doi:10.1038/sj.bjc.6602535)
Supplement: Supplementary Data [file 92-6602535x1.doc]

**SUPPLEMENTARY DATA**

| **SUPPLEMENTAL TABLE I: GENES SIGNIFICANTLY**^1^ **REGULATED BY GASTRIN** | |  |  |  |  |
| --- | --- | --- | --- | --- | --- |
|  | | | | | |
|  | |  |  |  |  |
| **Symbol** | **Gene name** | **Accession^2^** | **Ratio^3^** | **n^4^** | **p-value** |
|  |  |  |  |  |  |
| *Up-regulated* |  |  |  |  |  |
| ATF4 | activating transcription factor 4 (tax-responsive enhancer element B67) | AA600217 | 1,63 | 6 | 0,034 |
| RRM2 | Ribonucleotide reductase M2 polypeptide | AA187351 | 1,59 | 6 | 0,036 |
| BTG1 | B-cell translocation gene 1, anti-proliferative | N70463 | 1,57 | 6 | 0,021 |
| LGALS3BP | lectin, galactoside-binding, soluble, 3 binding protein (galectin 6 binding protein) | AA485353 | 1,54 | 6 | 0,002 |
| MAP1B | microtubule-associated protein 1B | AA219045 | 1,54 | 6 | 0,016 |
| CCNI | cyclin I | AA434408 | 1,52 | 6 | 0,005 |
| CEBPD | CCAAT/enhancer binding protein (C/EBP), delta | AA043506 | 1,51 | 5 | 0,021 |
| LAMR1 | laminin receptor 1 (67kD, ribosomal protein SA) | AA629897 | 1,49 | 6 | 0,037 |
| ANXA11 | annexin A11 | AA465051 | 1,47 | 6 | 0,024 |
| IL8 | interleukin 8 | AA102526 | 1,46 | 6 | 0,02 |
| EGR3 | early growth response 3 | R39111 | 1,45 | 5 | 0,013 |
| P23 | unactive progesterone receptor, 23 kD | AA669341 | 1,44 | 6 | 0,039 |
| ERCC5 | excision repair cross-complementing rodent repair deficiency, complementation group 5 (xeroderma pigmentosum, complementation group G (Cockayne syndrome)) | N62586 | 1,44 | 3 | 0,014 |
| HSAPO-MUCN | Apomucin | AA159577 | 1,44 | 4 | 0,007 |
| SATB1 | special AT-rich sequence binding protein 1 (binds to nuclear matrix/scaffold-associating DNA's) | AA022561 | 1,44 | 5 | 0,002 |
| HSRTS-BETA | rTS beta protein | N66132 | 1,42 | 6 | 0,024 |
| KRT15 | keratin 15 | AA878048 | 1,42 | 4 | 0,003 |
| EEF2 | eukaryotic translation elongation factor 2 | R20379 | 1,41 | 6 | 0,031 |
| ACADVL | acyl-Coenzyme A dehydrogenase, very long chain | AA464163 | 1,38 | 6 | 0,043 |
| GTF2F1 | general transcription factor IIF, polypeptide 1 (74kD subunit) | AA282092 | 1,37 | 5 | 0,043 |
| PCSK1 | proprotein convertase subtilisin/kexin type 1 | R17394 | 1,37 | 6 | 0,035 |
| RCN2 | reticulocalbin 2, EF-hand calcium binding domain | AA598676 | 1,36 | 4 | 0,022 |
| HLA-C | major histocompatibility complex, class I, C | AA464246 | 1,36 | 6 | 0,013 |
| MCM3 | Minichromosome maintenance deficient (S. cerevisiae) 3 | AA455786 | 1,35 | 6 | 0,017 |
| CCT6A | chaperonin containing TCP1, subunit 6A (zeta 1) | AA872690 | 1,35 | 4 | 0,047 |
| YWHAE | tyrosine 3-monooxygenase/tryptophan 5-monooxygenase activation protein, epsilon polypeptide | N21624 | 1,33 | 5 | 0,009 |
| HOXA1 | homeo box A1 | AA173290 | 1,33 | 6 | 0,042 |
| CAMP | cathelicidin antimicrobial peptide | AA609759 | 1,32 | 6 | 0,027 |
| CIN85 | c-Cbl-interacting protein | AA989257 | 1,31 | 6 | 0,006 |
| PPARG | peroxisome proliferative activated receptor, gamma | AA088517 | 1,31 | 4 | 0,032 |
| FGF1 | fibroblast growth factor 1 (acidic) | AA015793 | 1,3 | 6 | 0,025 |
| MLF2 | myeloid leukemia factor 2 | AA480835 | 1,3 | 6 | 0,033 |
| GLRX | glutaredoxin (thioltransferase) | AA291163 | 1,3 | 6 | 0,019 |
| NP | nucleoside phosphorylase | AA430382 | 1,29 | 6 | 0,025 |
| EWSR1 | Ewing sarcoma breakpoint region 1 | AA464184 | 1,29 | 6 | 0,019 |
| UBE3A | ubiquitin protein ligase E3A (human papilloma virus E6-associated protein, Angelman syndrome) | N94099 | 1,29 | 6 | 0,008 |
| S100A3 | S100 calcium-binding protein A3 | AA055242 | 1,29 | 6 | 0,021 |
| LMO2 | LIM domain only 2 (rhombotin-like 1) | AA464644 | 1,29 | 6 | 0,016 |
| SERPINA5 | serine (or cysteine) proteinase inhibitor, clade A (alpha-1 antiproteinase, antitrypsin), member 5 | AA858026 | 1,28 | 6 | 0,029 |
| BCAT2 | Branched chain aminotransferase 2, mitochondrial | AA436410 | 1,28 | 6 | 0,033 |
| PSME2 | proteasome (prosome, macropain) activator subunit 2 (PA28 beta) | H65395 | 1,28 | 6 | 0,036 |
| TRIP13 | thyroid hormone receptor interactor 13 | AA630784 | 1,27 | 6 | 0,033 |
| ETR101 | immediate early protein | AA496359 | 1,27 | 6 | 0,006 |
| EIF3S6 | eukaryotic translation initiation factor 3, subunit 6 (48kD) | AA669674 | 1,27 | 6 | 0,011 |
| BAK1 | BCL2-antagonist/killer 1 | H52673 | 1,26 | 6 | 0,003 |
| TFRC | transferrin receptor (p90, CD71) | AA488721 | 1,26 | 6 | 0,034 |
| LRMP | Lymphoid-restricted membrane protein | AA457051 | 1,26 | 6 | 0,047 |
| CAMK2D | calcium/calmodulin-dependent protein kinase (CaM kinase) II delta | AA283023 | 1,26 | 5 | 0,03 |
| RAD23A | RAD23 (S. cerevisiae) homolog A | AA476274 | 1,26 | 6 | 0,028 |
| NFIL3 | nuclear factor, interleukin 3 regulated | AA633811 | 1,26 | 6 | 0,024 |
| STK12 | serine/threonine kinase 12 | AA071486 | 1,26 | 6 | 0,017 |
| CDH6 | Cadherin 6, type 2, K-cadherin (fetal kidney) | AA421819 | 1,25 | 5 | 0,001 |
| GSTT1 | glutathione S-transferase theta 1 | H99813 | 1,25 | 6 | 0,021 |
| SIAT4C | Sialyltransferase 4C (beta-galactosidase alpha-2,3-sialytransferase) | AA453898 | 1,25 | 6 | 0,049 |
|  |  |  |  |  |  |
| *Down-regulated* | |  |  |  |  |
| FHL1 | four and a half LIM domains 1 | AA456394 | 0,48 | 6 | 0,002 |
| IL2RB | interleukin 2 receptor, beta | AA057156 | 0,48 | 6 | 0,01 |
| KIAA0421 | KIAA0421 protein | H98694 | 0,49 | 6 | 0,013 |
| MIG | monokine induced by gamma interferon | AA131406 | 0,53 | 5 | 0,027 |
| GNRH1 | gonadotropin-releasing hormone 1 (leutinizing-releasing hormone) | AA043996 | 0,55 | 6 | 0,047 |
| SPOP | speckle-type POZ protein | AA256459 | 0,59 | 6 | 0,041 |
| G6PD | glucose-6-phosphate dehydrogenase | AA424937 | 0,61 | 6 | 0,007 |
| LIG3 | ligase III, DNA, ATP-dependent | AA149292 | 0,61 | 5 | 0,023 |
| CCR1 | chemokine (C-C motif) receptor 1 | AA036881 | 0,63 | 5 | 0,001 |
| SYK | spleen tyrosine kinase | AA598572 | 0,63 | 4 | 0,007 |
| MGST1 | microsomal glutathione S-transferase 1 | AA495936 | 0,64 | 6 | 0,01 |
| CHC1L | chromosome condensation 1-like | AA495766 | 0,66 | 3 | 0,026 |
| EPS15 | epidermal growth factor receptor pathway substrate 15 | AA490223 | 0,66 | 6 | 0,013 |
| CDH11 | Cadherin 11, type 2, OB-cadherin (osteoblast) | AA136983 | 0,66 | 6 | 0,007 |
| IGFBP1 | insulin-like growth factor binding protein 1 | AA233079 | 0,66 | 6 | 0,041 |
| LTBR | lymphotoxin beta receptor (TNFR superfamily, member 3) | AA454646 | 0,67 | 6 | 0,019 |
| LBC | Lymphoid blast crisis oncogene | AA156936 | 0,7 | 3 | 0,042 |
| UBE2E1 | ubiquitin-conjugating enzyme E2E 1 (homologous to yeast UBC4/5) | AA044025 | 0,7 | 6 | 0,007 |
| PFKM | phosphofructokinase, muscle | AA099169 | 0,7 | 6 | 0,009 |
| SCYA2 | small inducible cytokine A2 (monocyte chemotactic protein 1, homologous to mouse Sig-je) | AA425102 | 0,71 | 6 | 0,011 |
| IL1RL1 | interleukin 1 receptor-like 1 | AA128153 | 0,72 | 6 | 0,037 |
| HDAC2 | histone deacetylase 2 | AA127093 | 0,72 | 6 | 0,031 |
| PSMD2 | proteasome (prosome, macropain) 26S subunit, non-ATPase, 2 | AA455193 | 0,74 | 4 | 0,022 |
| PIM1 | pim-1 oncogene | AA453663 | 0,75 | 5 | 0,024 |
| LOC51177 | CK2 interacting protein 1; HQ0024c protein | AA490216 | 0,76 | 6 | 0,017 |
| MGC5521 | hypothetical protein MGC5521 | AA454625 | 0,76 | 6 | 0,002 |
| LAMA5 | laminin, alpha 5 | AA459519 | 0,77 | 6 | 0,025 |
| HCLS1 | hematopoietic cell-specific Lyn substrate 1 | AA424575 | 0,77 | 6 | 0,035 |
| ITGAV | integrin, alpha V (vitronectin receptor, alpha polypeptide, antigen CD51) | AA029934 | 0,77 | 6 | 0,005 |
| PDE8A | phosphodiesterase 8A | AA233564 | 0,77 | 6 | 0,011 |
| MEL | mel transforming oncogene (derived from cell line NK14)- RAB8 homolog | AA064715 | 0,77 | 3 | 0,029 |
| SCYA16 | small inducible cytokine subfamily A (Cys-Cys), member 16 | T58775 | 0,77 | 6 | 0,038 |
| OS-9 | amplified in osteosarcoma | AA013336 | 0,77 | 6 | 0,03 |
| APOC3 | apolipoprotein C-III | N53169 | 0,78 | 6 | 0,032 |
| HOXD4 | homeo box D4 | AA447692 | 0,78 | 6 | 0,027 |
| MMP7 | matrix metalloproteinase 7 (matrilysin, uterine) | AA031513 | 0,78 | 6 | 0,015 |
| OR2A19 | Olfactory receptor, family 2, subfamily A, member 19 | AA962054 | 0,78 | 4 | 0,043 |
| RAB18 | RAB18, member RAS oncogene family | AA156821 | 0,79 | 6 | 0,033 |
| MAN2C1 | mannosidase, alpha, class 2C, member 1 | H45455 | 0,79 | 6 | 0,04 |
| GSTA4 | glutathione S-transferase A4 | AA152347 | 0,79 | 6 | 0,019 |
| ACAA2 | acetyl-Coenzyme A acyltransferase 2 (mitochondrial 3-oxoacyl-Coenzyme A thiolase) | H07926 | 0,79 | 6 | 0,002 |
|  |  |  |  |  |  |
| ^1^Significantly (p ≤ 0.05) differentially expressed genes with a microarray ratio < 0.8 or > 1.25. - ^2^GeneBank accession number. – ^3^Microarray ratio (treated/untreated cells). – ^4^Number of observations of each gene (one measured spot = one observation; since each probe was printed twice on each array, one hybridization may give two observations for each gene). The data are based on samples from two biological experiments of which one sample was hybridized once, and the other twice. | | | | | |

| **SUPPLEMENTAL TABLE II: GENES SIGNIFICANTLY^1^ REGULATED BY HGF** | | |  |  |  |
| --- | --- | --- | --- | --- | --- |
| **Symbol** | **Gene name** | **Accession^2^** | **Ratio^3^** | **n^4^** | **p-value** |
| *Up-regulated* |  |  |  |  |  |
| ELK4 | ELK4, ETS-domain protein (SRF accessory protein 1) | H61758 | 1,55 | 7 | 0,024 |
| CEBPD | CCAAT/enhancer binding protein (C/EBP), delta | AA043506 | 1,55 | 7 | 0,01 |
| MAT2A | methionine adenosyltransferase II, alpha | T59286 | 1,53 | 6 | 0,04 |
| ACTG2 | actin, gamma 2, smooth muscle, enteric | T60048 | 1,52 | 8 | 0,016 |
| NCOA3 | nuclear receptor coactivator 3 | AA156793 | 1,48 | 8 | 0,001 |
| ATF4 | activating transcription factor 4 (tax-responsive enhancer element B67) | AA600217 | 1,46 | 8 | 0,005 |
| LGALS3BP | lectin, galactoside-binding, soluble, 3 binding protein (galectin 6 binding protein) | AA485353 | 1,45 | 8 | 0,001 |
| GTF2F1 | general transcription factor IIF, polypeptide 1 (74kD subunit) | AA282092 | 1,45 | 8 | 0,008 |
| CCNI | cyclin I | AA434408 | 1,42 | 7 | 0,002 |
| LAMR1 | laminin receptor 1 (67kD, ribosomal protein SA) | AA629897 | 1,41 | 8 | 0,001 |
| MLF2 | Myeloid leukemia factor 2 | AA480835 | 1,4 | 8 | 0,001 |
| MAPK4 | Mitogen-activated protein kinase 4 | AA401035 | 1,4 | 8 | 0,003 |
| CYP2C8 | cytochrome P450, subfamily IIC (mephenytoin 4-hydroxylase), polypeptide 8 | N53136 | 1,39 | 8 | 0,018 |
| NFE2L1 | nuclear factor (erythroid-derived 2)-like 1 | AA496576 | 1,38 | 8 | <0.001 |
| MCM3 | minichromosome maintenance deficient (S. cerevisiae) 3 | AA455786 | 1,38 | 8 | 0,001 |
| CAMP | cathelicidin antimicrobial peptide | AA609759 | 1,36 | 8 | 0,001 |
| NFATC1 | nuclear factor of activated T-cells, cytoplasmic, calcineurin-dependent 1 | AA679278 | 1,36 | 7 | 0,011 |
| MYPT2 | myosin phosphatase, target subunit 2 | AA463926 | 1,36 | 7 | 0,004 |
| GSTT1 | glutathione S-transferase theta 1 | H99813 | 1,36 | 8 | 0,004 |
| HSRTSBETA | rTS beta protein | N66132 | 1,35 | 8 | 0,002 |
| ANXA4 | Annexin A4 | AA419108 | 1,35 | 6 | 0,034 |
| ACADVL | acyl-Coenzyme A dehydrogenase, very long chain | AA464163 | 1,33 | 8 | 0,001 |
| CSDA | cold shock domain protein A | AA465019 | 1,33 | 4 | 0,03 |
| PPIG | Peptidyl-prolyl isomerase G (cyclophilin G) | AA458502 | 1,33 | 8 | 0,008 |
| BCKDHA | branched chain keto acid dehydrogenase E1, alpha polypeptide (maple syrup urine disease) | AA477298 | 1,33 | 8 | 0,019 |
| MAP1B | microtubule-associated protein 1B | AA219045 | 1,33 | 8 | 0,001 |
| IGF2R | insulin-like growth factor 2 receptor | T62547 | 1,32 | 8 | 0,028 |
| LMO2 | LIM domain only 2 (rhombotin-like 1) | AA464644 | 1,32 | 8 | 0,002 |
| GSTM5 | glutathione S-transferase M5 | AA056232 | 1,31 | 8 | 0,004 |
| NP | nucleoside phosphorylase | AA430382 | 1,3 | 8 | 0,002 |
| DSP | desmoplakin (DPI, DPII) | H90899 | 1,3 | 7 | 0,031 |
| BAK1 | BCL2-antagonist/killer 1 | H52673 | 1,29 | 8 | 0,017 |
| STK12 | serine/threonine kinase 12 | AA071486 | 1,28 | 8 | 0,006 |
| TJ6 | TJ6 protein | N70122 | 1,28 | 7 | 0,003 |
| ANXA11 | Annexin A11 | AA465051 | 1,27 | 8 | 0,023 |
| DPYD | dihydropyrimidine dehydrogenase | AA428170 | 1,27 | 8 | 0,008 |
| PCDH1 | protocadherin 1 (cadherin-like 1) | AA443557 | 1,27 | 8 | <0.001 |
| SYPL | synaptophysin-like protein | AA430698 | 1,27 | 8 | 0,004 |
| PDGFRA | platelet-derived growth factor receptor, alpha polypeptide | H23235 | 1,27 | 8 | 0,019 |
| CDC6 | CDC6 (cell division cycle 6, S. cerevisiae) homolog | H59203 | 1,26 | 8 | 0,008 |
| CIN85 | c-Cbl-interacting protein | AA989257 | 1,26 | 8 | 0,008 |
| HLA-C | major histocompatibility complex, class I, C | AA464246 | 1,26 | 8 | 0,002 |
| MRPS21 | mitochondrial ribosomal protein S21 | AA443497 | 1,26 | 8 | 0,002 |
| MDH1 | malate dehydrogenase 1, NAD (soluble) | AA403295 | 1,26 | 8 | 0,017 |
| GSK3B | Glycogen synthase kinase 3 beta | R93911 | 1,26 | 7 | 0,041 |
| ACTN3 | actinin, alpha 3 | AA196000 | 1,25 | 8 | 0,044 |
| COL1A2 | *Collagen, type I, alpha 2* | AA490172 | 1,25 | 6 | 0,026 |
|  |  |  |  |  |  |
| *Down-regulated* | |  |  |  |  |
| IL2RB | interleukin 2 receptor, beta | AA057156 | 0,46 | 8 | 0,005 |
| PIM1 | pim-1 oncogene | AA453663 | 0,49 | 6 | 0,035 |
| CD164 | CD164 antigen, sialomucin | AA598561 | 0,55 | 8 | 0,01 |
| TIMP3 | tissue inhibitor of metalloproteinase 3 (Sorsby fundus dystrophy, pseudoinflammatory) | AA099153 | 0,56 | 8 | 0,018 |
| GNRH1 | gonadotropin-releasing hormone 1 (leutinizing-releasing hormone) | AA043996 | 0,57 | 8 | 0,011 |
| FHL1 | four and a half LIM domains 1 | AA456394 | 0,59 | 8 | 0,021 |
| CCR1 | chemokine (C-C motif) receptor 1 | AA036881 | 0,6 | 8 | 0,021 |
| TRAF6 | TNF receptor-associated factor 6 | AA456692 | 0,6 | 8 | 0,028 |
| CGI-204 | CGI-204 protein | AA410394 | 0,61 | 8 | 0,005 |
| KIAA0421 | KIAA0421 protein | H98694 | 0,62 | 8 | 0,019 |
| EPS15 | epidermal growth factor receptor pathway substrate 15 | AA490223 | 0,63 | 8 | 0,001 |
| CDH11 | Cadherin 11, type 2, OB-cadherin (osteoblast) | AA136983 | 0,65 | 8 | 0,001 |
| PFKM | phosphofructokinase, muscle | AA099169 | 0,66 | 8 | 0,001 |
| MIG | monokine induced by gamma interferon | AA131406 | 0,66 | 8 | 0,011 |
| BDNF | brain-derived neurotrophic factor | AA262988 | 0,66 | 8 | 0,015 |
| FRDA | Friedreich ataxia | AA253413 | 0,67 | 8 | 0,035 |
| ALAS1 | aminolevulinate, delta-, synthase 1 | AA453691 | 0,67 | 8 | 0,02 |
| TNFRSF11B | tumor necrosis factor receptor superfamily, member 11b (osteoprotegerin) | AA194983 | 0,67 | 8 | 0,001 |
| ADA | adenosine deaminase | AA683578 | 0,67 | 8 | 0,029 |
| FCGR2A | Fc fragment of IgG, low affinity IIa, receptor for (CD32) | AA634109 | 0,68 | 8 | 0,003 |
| SNRPB | small nuclear ribonucleoprotein polypeptides B and B1 | AA599116 | 0,69 | 8 | 0,047 |
| NID2 | Nidogen 2 | AA479199 | 0,69 | 8 | 0,007 |
| GTF2B | general transcription factor IIB | H23978 | 0,69 | 8 | 0,046 |
| SCYA16 | small inducible cytokine subfamily A (Cys-Cys), member 16 | T58775 | 0,7 | 8 | 0,002 |
| SPOP | speckle-type POZ protein | AA256459 | 0,7 | 8 | 0,001 |
| MEIS2 | Meis (mouse) homolog 2 | AA148641 | 0,71 | 8 | 0,013 |
| UBE2E1 | ubiquitin-conjugating enzyme E2E 1 (homologous to yeast UBC4/5) | AA044025 | 0,72 | 8 | 0,001 |
| ACAA2 | acetyl-Coenzyme A acyltransferase 2 (mitochondrial 3-oxoacyl-Coenzyme A thiolase) | H07926 | 0,72 | 8 | 0,031 |
| LAMA5 | laminin, alpha 5 | AA459519 | 0,72 | 8 | 0,021 |
| SDC4 | Syndecan 4 (amphiglycan, ryudocan) | AA148736 | 0,72 | 7 | 0,047 |
| TCEB1L | transcription elongation factor B (SIII), polypeptide 1-like | AA136533 | 0,73 | 8 | 0,047 |
| UVRAG | UV radiation resistance associated gene | AA490771 | 0,73 | 8 | 0,025 |
| INPP5A | inositol polyphosphate-5-phosphatase, 40kD | T58773 | 0,73 | 7 | 0,008 |
| MGST1 | microsomal glutathione S-transferase 1 | AA495936 | 0,73 | 8 | 0,045 |
| HMOX1 | heme oxygenase (decycling) 1 | T71757 | 0,73 | 8 | 0,025 |
| JUND | jun D proto-oncogene | AA418670 | 0,74 | 8 | 0,015 |
| COL4A5 | Collagen, type IV, alpha 5 (Alport syndrome) | AA029997 | 0,74 | 8 | 0,023 |
| LOC51042 | zinc finger protein | AA033532 | 0,74 | 5 | 0,033 |
| CD58 | CD58 antigen, (lymphocyte function-associated antigen 3) | AA136359 | 0,75 | 8 | 0,015 |
| GEM | GTP-binding protein overexpressed in skeletal muscle | AA418077 | 0,75 | 8 | 0,028 |
| RAB18 | RAB18, member RAS oncogene family | AA156821 | 0,75 | 8 | 0,003 |
| TSC22 | transforming growth factor beta-stimulated protein TSC-22 | AA664389 | 0,75 | 6 | 0,014 |
| LOC51177 | CK2 interacting protein 1; HQ0024c protein | AA490216 | 0,76 | 8 | 0,028 |
| WT1 | Wilms tumor 1 | AA130187 | 0,76 | 8 | 0,046 |
| KIAA0203 | KIAA0203 gene product | AA047435 | 0,76 | 8 | 0,009 |
| HCLS1 | hematopoietic cell-specific Lyn substrate 1 | AA424575 | 0,77 | 7 | 0,041 |
| EIF2S2 | eukaryotic translation initiation factor 2, subunit 2 (beta, 38kD ) | AA027240 | 0,77 | 8 | 0,025 |
| SIAH2 | seven in absentia (Drosophila) homolog 2 | AA029041 | 0,77 | 7 | 0,003 |
| STATI2 | STAT induced STAT inhibitor-2 | AA137031 | 0,78 | 8 | 0,01 |
| ITGAV | integrin, alpha V (vitronectin receptor, alpha polypeptide, antigen CD51) | AA029934 | 0,78 | 8 | 0,001 |
| TOB1 | transducer of ERBB2, 1 | AA490213 | 0,79 | 8 | 0,005 |
| CX3CR1 | chemokine (C-X3-C) receptor 1 | N51278 | 0,8 | 8 | 0,005 |
| BIRC1 | baculoviral IAP repeat-containing 1 | AA621150 | 0,8 | 8 | 0,022 |
| CD163 | CD163 antigen | AA401693 | 0,8 | 8 | 0,015 |
|  | | | | | |
| ^1^Significantly (p ≤ 0.05) differentially expressed genes with a microarray ratio < 0.8 or > 1.25. - ^2^GeneBank accession number. – ^3^Microarray ratio (treated/untreated cells). – ^4^Number of observations of each gene (one measured spot = one observation; since each probe was printed twice on each array, one hybridization may give two observations for each gene). The data are based on samples from two biological experiments which both were hybridized twice. | | | | | |
|  |  |  |  |  |  |

| **SUPPLEMENTAL TABLE III: GENES SIGNIFICANTLY^1^ REGULATED BY EGF** | | |  |  |  |
| --- | --- | --- | --- | --- | --- |
| **Symbol** | **Gene name** | **Accession^2^** | **Ratio^3^** | **n^4^** | **p-value** |
| *Up-regulated* |  |  |  |  |  |
| TR | thioredoxin reductase beta | AA434130 | 1,56 | 4 | 0,013 |
| CAMP | cathelicidin antimicrobial peptide | AA609759 | 1,5 | 4 | 0,041 |
| PPIG | Peptidyl-prolyl isomerase G (cyclophilin G) | AA458502 | 1,49 | 4 | 0,046 |
| ATF4 | activating transcription factor 4 (tax-responsive enhancer element B67) | AA600217 | 1,47 | 4 | 0,003 |
| DYT1 | Dystonia 1, torsion (autosomal dominant; torsin A) | AA394148 | 1,44 | 3 | 0,026 |
| DPYD | dihydropyrimidine dehydrogenase | AA428170 | 1,44 | 3 | 0,008 |
| NF2 | neurofibromin 2 (bilateral acoustic neuroma) | AA428960 | 1,44 | 4 | 0,002 |
| CYP2C8 | cytochrome P450, subfamily IIC (mephenytoin 4-hydroxylase), polypeptide 8 | N53136 | 1,44 | 4 | 0,003 |
| DSP | desmoplakin (DPI, DPII) | H90899 | 1,43 | 4 | 0,024 |
| NFIL3 | nuclear factor, interleukin 3 regulated | AA633811 | 1,4 | 4 | 0,043 |
| ACTN3 | actinin, alpha 3 | AA196000 | 1,4 | 4 | 0,008 |
| TRD@ | T cell receptor delta locus | AA670107 | 1,38 | 4 | 0,002 |
| LMO7 | LIM domain only 7 | AA005112 | 1,36 | 4 | 0,004 |
| CDH6 | Cadherin 6, type 2, K-cadherin (fetal kidney) | AA421819 | 1,36 | 4 | 0,031 |
| ACY1 | aminoacylase 1 | AA402915 | 1,36 | 4 | <0.001 |
| PCCA | propionyl Coenzyme A carboxylase, alpha polypeptide | AA608575 | 1,36 | 4 | 0,027 |
| NFE2L1 | nuclear factor (erythroid-derived 2)-like 1 | AA496576 | 1,35 | 4 | 0,037 |
| ERCC5 | excision repair cross-complementing rodent repair deficiency, complementation group 5 (xeroderma pigmentosum, complementation group G (Cockayne syndrome)) | N62586 | 1,35 | 4 | 0,037 |
| RBBP1 | retinoblastoma-binding protein 1 | AA128328 | 1,35 | 4 | 0,028 |
| FLJ20275 | hypothetical protein FLJ20275 | AA458682 | 1,34 | 4 | 0,033 |
| KIAA0135 | KIAA0135 protein | AA427740 | 1,34 | 4 | 0,028 |
| SATB1 | special AT-rich sequence binding protein 1 (binds to nuclear matrix/scaffold-associating DNA's) | AA022561 | 1,33 | 4 | 0,007 |
| VDAC1 | voltage-dependent anion channel 1 | AA044059 | 1,32 | 4 | 0,029 |
| S100A2 | S100 calcium-binding protein A2 | AA458884 | 1,31 | 4 | 0,034 |
| SLAM | signaling lymphocytic activation molecule | AA458996 | 1,3 | 4 | 0,021 |
| IRAK1 | interleukin-1 receptor-associated kinase 1 | AA683550 | 1,29 | 4 | 0,019 |
| SERPINB3 | serine (or cysteine) proteinase inhibitor, clade B (ovalbumin), member 3 | AA398883 | 1,29 | 4 | 0,018 |
| CIN85 | c-Cbl-interacting protein | AA989257 | 1,29 | 4 | 0,031 |
| IMPA2 | inositol(myo)-1(or 4)-monophosphatase 2 | R42685 | 1,28 | 4 | 0,047 |
| NEK1 | NIMA (never in mitosis gene a)-related kinase 1 | N71695 | 1,28 | 4 | 0,045 |
| NP | nucleoside phosphorylase | AA430382 | 1,27 | 4 | 0,008 |
| LMO2 | LIM domain only 2 (rhombotin-like 1) | AA464644 | 1,27 | 4 | 0,05 |
| FLJ22418 | hypothetical protein FLJ22418 | AA404609 | 1,27 | 4 | 0,009 |
| CPA3 | carboxypeptidase A3 (mast cell) | T64223 | 1,26 | 4 | 0,026 |
| TYROBP | TYRO protein tyrosine kinase binding protein | H12338 | 1,25 | 4 | 0,013 |
| MADH7 | MAD (mothers against decapentaplegic, Drosophila) homolog 7 | R82176 | 1,25 | 4 | 0,021 |
| GSTT1 | glutathione S-transferase theta 1 | H99813 | 1,25 | 4 | 0,041 |
|  |  |  |  |  |  |
| *Down-regulated* | |  |  |  |  |
| IL2RB | interleukin 2 receptor, beta | AA057156 | 0,36 | 4 | 0,021 |
| CD164 | CD164 antigen, sialomucin | AA598561 | 0,44 | 4 | 0,004 |
| FHL1 | four and a half LIM domains 1 | AA456394 | 0,46 | 4 | 0,02 |
| GNRH1 | gonadotropin-releasing hormone 1 (leutinizing-releasing hormone) | AA043996 | 0,47 | 4 | 0,005 |
| TIMP3 | tissue inhibitor of metalloproteinase 3 (Sorsby fundus dystrophy, pseudoinflammatory) | AA099153 | 0,47 | 3 | 0,008 |
| CCR1 | chemokine (C-C motif) receptor 1 | AA036881 | 0,53 | 4 | 0,036 |
| MEIS2 | Meis (mouse) homolog 2 | AA148641 | 0,53 | 4 | 0,005 |
| NID2 | Nidogen 2 | AA479199 | 0,55 | 4 | 0,028 |
| SPOP | speckle-type POZ protein | AA256459 | 0,55 | 4 | 0,016 |
| HCK | hemopoietic cell kinase | AA149096 | 0,56 | 4 | 0,01 |
| PFKM | phosphofructokinase, muscle | AA099169 | 0,59 | 4 | 0,017 |
| ELF2 | E74-like factor 2 (ets domain transcription factor) | AA453714 | 0,6 | 4 | 0,006 |
| LAMB2 | laminin, beta 2 (laminin S) | AA156802 | 0,61 | 4 | 0,012 |
| KIAA0203 | KIAA0203 gene product | AA047435 | 0,61 | 4 | 0,026 |
| TDG | Thymine-DNA glycosylase | AA490546 | 0,61 | 3 | 0,003 |
| MMP7 | matrix metalloproteinase 7 (matrilysin, uterine) | AA031513 | 0,62 | 4 | 0,018 |
| BDNF | brain-derived neurotrophic factor | AA262988 | 0,62 | 4 | 0,032 |
| LTBR | lymphotoxin beta receptor (TNFR superfamily, member 3) | AA454646 | 0,62 | 4 | 0,01 |
| CDH11 | Cadherin 11, type 2, OB-cadherin (osteoblast) | AA136983 | 0,64 | 4 | 0,019 |
| PIM1 | pim-1 oncogene | AA453663 | 0,66 | 3 | 0,022 |
| ZNF83 | zinc finger protein 83 (HPF1) | AA043743 | 0,66 | 4 | 0,027 |
| NFIX | nuclear factor I/X (CCAAT-binding transcription factor) | AA406269 | 0,66 | 4 | 0,013 |
| HMOX1 | heme oxygenase (decycling) 1 | T71757 | 0,66 | 4 | 0,005 |
| TGFBR3 | transforming growth factor, beta receptor III (betaglycan, 300kD) | H62473 | 0,67 | 4 | 0,003 |
| HDAC2 | histone deacetylase 2 | AA127093 | 0,67 | 4 | 0,014 |
| COL4A5 | Collagen, type IV, alpha 5 (Alport syndrome) | AA029997 | 0,68 | 4 | 0,016 |
| HOXD4 | homeo box D4 | AA447692 | 0,69 | 4 | 0,018 |
| EPS15 | epidermal growth factor receptor pathway substrate 15 | AA490223 | 0,69 | 4 | 0,002 |
| SCYA16 | small inducible cytokine subfamily A (Cys-Cys), member 16 | T58775 | 0,71 | 4 | 0,013 |
| GSTA4 | glutathione S-transferase A4 | AA152347 | 0,73 | 4 | 0,036 |
| COPS5 | COP9 (constitutive photomorphogenic, Arabidopsis, homolog) subunit 5 | AA460599 | 0,73 | 4 | 0,012 |
| LAMA5 | laminin, alpha 5 | AA459519 | 0,73 | 4 | 0,005 |
| LY6E | lymphocyte antigen 6 complex, locus E | AA865464 | 0,74 | 4 | 0,031 |
| MAN2C1 | mannosidase, alpha, class 2C, member 1 | H45455 | 0,74 | 4 | 0,031 |
| CA150 | transcription factor CA150 | AA045180 | 0,74 | 4 | 0,014 |
| SYK | spleen tyrosine kinase | AA598572 | 0,75 | 3 | 0,043 |
| PPY | pancreatic polypeptide | AA844998 | 0,75 | 4 | 0,001 |
| IGSF3 | immunoglobulin superfamily, member 3 | AI002566 | 0,76 | 4 | 0,04 |
| INPP5A | inositol polyphosphate-5-phosphatase, 40kD | T58773 | 0,76 | 4 | 0,016 |
| FLJ20312 | hypothetical protein FLJ20312 | AA633957 | 0,76 | 4 | 0,004 |
| PLD2 | phospholipase D2 | AA418524 | 0,77 | 4 | 0,019 |
| TAF3B2 | TATA box binding protein (TBP)-associated factor, RNA polymerase III, GTF3B subunit 2 | AA453787 | 0,77 | 4 | 0,017 |
| STAT4 | signal transducer and activator of transcription 4 | R91570 | 0,77 | 4 | 0,025 |
| THOP1 | thimet oligopeptidase 1 | AA829295 | 0,77 | 4 | 0,04 |
| PLD1 | phospholipase D1, phophatidylcholine-specific | AA626014 | 0,77 | 3 | 0,019 |
| RBM5 | RNA binding motif protein 5 | W73892 | 0,77 | 4 | 0,005 |
| SFRS9 | Splicing factor, arginine/serine-rich 9 | AA490721 | 0,77 | 4 | 0,003 |
| RHCG | Rh type C glycoprotein | AA864183 | 0,78 | 4 | 0,01 |
| JUND | jun D proto-oncogene | AA418670 | 0,78 | 4 | 0,032 |
| PSMA1 | proteasome (prosome, macropain) subunit, alpha type, 1 | R27585 | 0,78 | 4 | 0,001 |
| CEACAM1 | carcinoembryonic antigen-related cell adhesion molecule 1 (biliary glycoprotein) | AA411757 | 0,78 | 3 | 0,029 |
| CYP4A11 | cytochrome P450, subfamily IVA, polypeptide 11 | W84868 | 0,79 | 4 | 0,009 |
| HCS | cytochrome c | AA865265 | 0,79 | 4 | 0,011 |
| SAS | Sarcoma amplified sequence | AA664211 | 0,79 | 4 | 0,024 |
| UBE2B | ubiquitin-conjugating enzyme E2B (RAD6 homolog) | AA598492 | 0,79 | 4 | 0,034 |
| CPA1 | carboxypeptidase A1 (pancreatic) | AA845178 | 0,79 | 4 | 0,006 |
| UBE2E1 | ubiquitin-conjugating enzyme E2E 1 (homologous to yeast UBC4/5) | AA044025 | 0,79 | 4 | 0,011 |
| CD58 | CD58 antigen, (lymphocyte function-associated antigen 3) | AA136359 | 0,8 | 4 | 0,024 |
| SPINT2 | serine protease inhibitor, Kunitz type, 2 | AA459039 | 0,8 | 4 | 0,012 |
| IFI35 | interferon-induced protein 35 | AA827287 | 0,8 | 4 | 0,014 |
| GPX3 | glutathione peroxidase 3 (plasma) | AA664180 | 0,8 | 4 | 0,024 |
| FABP3 | fatty acid binding protein 3, muscle and heart (mammary-derived growth inhibitor) | AA148548 | 0,8 | 4 | 0,019 |
|  |  |  |  |  |  |
| ^1^Significantly (p ≤ 0.05) differentially expressed genes with a microarray ratio < 0.8 or > 1.25. - ^2^GeneBank accession number. – ^3^Microarray ratio (treated/untreated cells). – ^4^Number of observations of each gene (one measured spot = one observation; since each probe was printed twice on each array, one hybridization may give two observations for each gene). The data are based on samples from one biological experiment which was hybridized twice. | | | | | |

| **SUPPLEMENTAL TABLE IV: GENES SIGNIFICANTLY^1^ REGULATED BY PACAP** | | | |  |  |
| --- | --- | --- | --- | --- | --- |
| **Symbol** | **Gene name** | **Accession^2^** | **Ratio^3^** | **n^4^** | **p-value** |
| *Up-regulated* |  |  |  |  |  |
| BTG1 | B-cell translocation gene 1, anti-proliferative | N70463 | 2,39 | 4 | 0,009 |
| ATF4 | Activating transcription factor 4 (tax-responsive enhancer element B67) | AA600217 | 2,16 | 4 | 0,027 |
| S100A3 | S100 calcium-binding protein A3 | AA055242 | 1,76 | 4 | 0,045 |
| GARS | glycyl-tRNA synthetase | AA629909 | 1,67 | 4 | 0,002 |
| RCN2 | Reticulocalbin 2, EF-hand calcium binding domain | AA598676 | 1,63 | 4 | 0,004 |
| TOP1 | Topoisomerase (DNA) I | AA232856 | 1,63 | 4 | 0,021 |
| IL8 | Interleukin 8 | AA102526 | 1,6 | 4 | 0,01 |
| CDKN2C | cyclin-dependent kinase inhibitor 2C (p18, inhibits CDK4) | N72115 | 1,57 | 4 | 0,012 |
| LMO7 | LIM domain only 7 | AA005112 | 1,57 | 4 | 0,035 |
| RRM2 | Ribonucleotide reductase M2 polypeptide | AA187351 | 1,47 | 4 | 0,009 |
| VDAC1 | voltage-dependent anion channel 1 | AA044059 | 1,46 | 4 | 0,032 |
| SFRS11 | Splicing factor, arginine/serine-rich 11 | H56944 | 1,46 | 4 | 0,007 |
| SFRS3 | Splicing factor, arginine/serine-rich 3 | AA598400 | 1,45 | 4 | 0,025 |
| SATB1 | special AT-rich sequence binding protein 1 (binds to nuclear matrix/scaffold-associating DNA's) | AA022561 | 1,44 | 4 | 0,036 |
| EGR3 | early growth response 3 | R39111 | 1,42 | 4 | 0,013 |
| MAPK4 | Mitogen-activated protein kinase 4 | AA401035 | 1,42 | 3 | 0,001 |
| ACTG2 | actin, gamma 2, smooth muscle, enteric | T60048 | 1,42 | 4 | 0,027 |
| DSP | Desmoplakin (DPI, DPII) | H90899 | 1,42 | 4 | 0,016 |
| YWHAE | tyrosine 3-monooxygenase/tryptophan 5-monooxygenase activation protein, epsilon polypeptide | N21624 | 1,41 | 4 | 0,012 |
| LOC54499 | Putative membrane protein | T98352 | 1,39 | 4 | 0,05 |
| ELK4 | ELK4, ETS-domain protein (SRF accessory protein 1) | H61758 | 1,39 | 4 | 0,007 |
| CCNE1 | cyclin E1 | T54121 | 1,38 | 4 | 0,028 |
| TMSB4X | Thymosin, beta 4, X chromosome | AA634103 | 1,37 | 4 | 0,011 |
| CDC37 | CDC37 (cell division cycle 37, S. cerevisiae, homolog) | AA458870 | 1,36 | 4 | 0,033 |
| NCOA3 | nuclear receptor coactivator 3 | AA156793 | 1,36 | 4 | 0,025 |
| MET | met proto-oncogene (hepatocyte growth factor receptor) | AA410591 | 1,35 | 4 | 0,036 |
| RPS16 | Ribosomal protein S16 | AA668301 | 1,35 | 4 | 0,007 |
| CDC10 | CDC10 (cell division cycle 10, S. cerevisiae, homolog) | AA633993 | 1,35 | 4 | 0,041 |
| PCCA | Propionyl Coenzyme A carboxylase, alpha polypeptide | AA608575 | 1,34 | 4 | 0,01 |
| KRT18 | keratin 18 | AA664179 | 1,34 | 4 | 0,022 |
| CLIC1 | Chloride intracellular channel 1 | AA486518 | 1,33 | 4 | 0,031 |
| CIN85 | c-Cbl-interacting protein | AA989257 | 1,32 | 4 | 0,01 |
| EDR2 | early development regulator 2 (homolog of polyhomeotic 2) | AA598840 | 1,32 | 4 | 0,039 |
| MGC8471 | Hypothetical protein MGC8471 | AA447502 | 1,32 | 4 | 0,032 |
| TEAD4 | TEA domain family member 4 | W74602 | 1,31 | 4 | 0,009 |
| ETR101 | Immediate early protein | AA496359 | 1,31 | 4 | 0,02 |
| HNRPDL | Heterogeneous nuclear ribonucleoprotein D-like | AA598578 | 1,31 | 4 | 0,014 |
| HSAPOMUCN | Apomucin | AA159577 | 1,31 | 3 | 0,01 |
| COPEB | core promoter element binding protein | AA055585 | 1,3 | 4 | 0,024 |
| PTE1 | Peroxisomal acyl-CoA thioesterase | AA447824 | 1,29 | 4 | 0,005 |
| EDN1 | Endothelin 1 | H11003 | 1,29 | 8 | 0,001 |
| LTA | Lymphotoxin alpha (TNF superfamily, member 1) | W72329 | 1,29 | 4 | 0,043 |
| ADORA2B | Adenosine A2b receptor | AA055350 | 1,28 | 4 | 0,023 |
| FOSB | FBJ murine osteosarcoma viral oncogene homolog B | T62179 | 1,27 | 4 | 0,02 |
| BMP4 | bone morphogenetic protein 4 | AA463225 | 1,25 | 4 | 0,001 |
| TCF20 | Transcription factor 20 (AR1) | AA857407 | 1,25 | 4 | 0,017 |
|  |  |  |  |  |  |
| *Down-regulated* | |  |  |  |  |
| IL2RB | Interleukin 2 receptor, beta | AA057156 | 0,44 | 3 | 0,014 |
| BDNF | brain-derived neurotrophic factor | AA262988 | 0,53 | 4 | 0,016 |
| PFKM | Phosphofructokinase, muscle | AA099169 | 0,59 | 4 | 0,003 |
| CDH11 | Cadherin 11, type 2, OB-cadherin (osteoblast) | AA136983 | 0,6 | 4 | 0,01 |
| TIMP3 | tissue inhibitor of metalloproteinase 3 (Sorsby fundus dystrophy, pseudoinflammatory) | AA099153 | 0,61 | 3 | 0,002 |
| UVRAG | UV radiation resistance associated gene | AA490771 | 0,63 | 4 | 0,019 |
| CD58 | CD58 antigen, (lymphocyte function-associated antigen 3) | AA136359 | 0,64 | 4 | 0,002 |
| LAMA5 | laminin, alpha 5 | AA459519 | 0,64 | 4 | 0,002 |
| LAMB2 | laminin, beta 2 (laminin S) | AA156802 | 0,64 | 4 | 0,017 |
| ELF2 | E74-like factor 2 (ets domain transcription factor) | AA453714 | 0,65 | 4 | 0,045 |
| CCR1 | Chemokine (C-C motif) receptor 1 | AA036881 | 0,65 | 4 | 0,034 |
| GADD34 | growth arrest and DNA-damage-inducible 34 | AA460168 | 0,65 | 4 | 0,023 |
| SNRPB | small nuclear ribonucleoprotein polypeptides B and B1 | AA599116 | 0,65 | 4 | 0,01 |
| TRAF6 | TNF receptor-associated factor 6 | AA456692 | 0,66 | 3 | 0,047 |
| D123 | D123 gene product | AA448289 | 0,67 | 4 | 0,019 |
| WT1 | Wilms tumor 1 | AA130187 | 0,68 | 4 | 0,033 |
| PLCD1 | Phospholipase C, delta 1 | R55490 | 0,69 | 4 | 0,005 |
| TCEB1L | Transcription elongation factor B (SIII), polypeptide 1-like | AA136533 | 0,7 | 4 | 0,004 |
| PIM1 | pim-1 oncogene | AA453663 | 0,7 | 4 | 0,035 |
| CLECSF2 | C-type (calcium dependent, carbohydrate-recognition domain) lectin, superfamily member 2 (activation-induced) | AA417921 | 0,72 | 4 | 0,018 |
| CPA1 | Carboxypeptidase A1 (pancreatic) | AA845178 | 0,73 | 4 | 0,042 |
| DAXX | death-associated protein 6 | N73287 | 0,73 | 4 | 0,014 |
| LTBP2 | latent transforming growth factor beta binding protein 2 | AA424629 | 0,74 | 4 | 0,041 |
| RBM5 | RNA binding motif protein 5 | W73892 | 0,74 | 4 | 0,047 |
| LOC51027 | CGI-143 protein | AA399245 | 0,74 | 4 | 0,027 |
| ACAA2 | acetyl-Coenzyme A acyltransferase 2 (mitochondrial 3-oxoacyl-Coenzyme A thiolase) | H07926 | 0,74 | 4 | 0,029 |
| PIK3C3 | Phosphoinositide-3-kinase, class 3 | AA455605 | 0,75 | 4 | 0,047 |
| CL683 | Hypothetical protein | AA418750 | 0,75 | 4 | 0,028 |
| KIAA0128 | KIAA0128 protein; septin 2 | R76772 | 0,76 | 4 | 0,026 |
| SLC25A6 | solute carrier family 25 (mitochondrial carrier; adenine nucleotide translocator), member 6 | W44701 | 0,76 | 4 | 0,011 |
| TGM2 | Transglutaminase 2 (C polypeptide, protein-glutamine-gamma-glutamyltransferase) | R97066 | 0,76 | 4 | 0,034 |
| TRIP8 | thyroid hormone receptor interactor 8 | AA425650 | 0,77 | 4 | 0,04 |
| NOH61 | Putative nucleolar RNA helicase | AA461476 | 0,77 | 4 | 0,004 |
| TGFBR2 | Transforming growth factor, beta receptor II (70-80kD) | AA487034 | 0,77 | 4 | 0,034 |
| SUPT5H | Suppressor of Ty (S.cerevisiae) 5 homolog | R21614 | 0,77 | 4 | 0,041 |
| PAK1 | p21/Cdc42/Rac1-activated kinase 1 (yeast Ste20-related) | AA890663 | 0,77 | 4 | 0,009 |
| ZNF83 | zinc finger protein 83 (HPF1) | AA043743 | 0,77 | 4 | 0,024 |
| FUBP1 | far upstream element (FUSE) binding protein 1 | AA676848 | 0,77 | 4 | 0,04 |
| EPRS | Glutamyl-prolyl-tRNA synthetase | AA599158 | 0,77 | 4 | 0,016 |
| SPINK1 | serine protease inhibitor, Kazal type 1 | AA845156 | 0,77 | 4 | 0,043 |
| UQCRFS1 | Ubiquinol-cytochrome c reductase, Rieske iron-sulfur polypeptide 1 | AA448184 | 0,78 | 4 | 0,005 |
| IFI35 | Interferon-induced protein 35 | AA827287 | 0,78 | 4 | 0,014 |
| ADAM17 | a disintegrin and metalloproteinase domain 17 (tumor necrosis factor, alpha, converting enzyme) | AA878951 | 0,78 | 4 | 0,033 |
| CGI-204 | CGI-204 protein | AA410394 | 0,78 | 4 | 0,023 |
| TSPAN-5 | Tetraspan 5 | AA464601 | 0,78 | 4 | 0,028 |
| NTKL | N-terminal kinase-like | AA447782 | 0,78 | 4 | 0,001 |
| SP1 | Sp1 transcription factor | N34776 | 0,79 | 4 | 0,036 |
| CLIC3 | Chloride intracellular channel 3 | N91135 | 0,79 | 4 | 0,012 |
| PIR | Pirin | H69334 | 0,8 | 4 | 0,046 |
| GPR30 | G protein-coupled receptor 30 | AA810225 | 0,8 | 4 | 0,038 |
|  |  |  |  |  |  |
| ^1^Significantly (p ≤ 0.05) differentially expressed genes with a microarray ratio < 0.8 or > 1.25. -^2^GeneBank accession number. – ^3^Microarray ratio (treated/untreated cells). – ^4^Number of observations of each gene (one measured spot = one observation; since each probe was printed twice on each array, one hybridization may give two observations for each gene). The data are based on samples from one biological experiment which was hybridized twice. | | | | | |
